# Positive and Purifying Selection Influence the Evolution of *Doublesex* in the *Anastrepha fraterculus* Species Group

**DOI:** 10.1371/journal.pone.0033446

**Published:** 2012-03-13

**Authors:** Iderval S. Sobrinho, Reinaldo A. de Brito

**Affiliations:** Departamento de Genética e Evolução, Universidade Federal de São Carlos, São Carlos, Brazil; University of Arkanas, United States of America

## Abstract

The gene *doublesex* (*dsx*) is considered to be under strong selective constraint along its evolutionary history because of its central role in somatic sex differentiation in insects. However, previous studies of *dsx* used global estimates of evolutionary rates to investigate its molecular evolution, which potentially miss signals of adaptive changes in generally conserved genes. In this work, we investigated the molecular evolution of *dsx* in the *Anastrepha fraterculus* species group (Diptera, Tephritidae), and test the hypothesis that this gene evolved solely by purifying selection using divergence-based and population-based methods. In the first approach, we compared sequences from *Anastrepha* and other Tephritidae with other Muscomorpha species, analyzed variation in nonsynonymous to synonymous rate ratios (*dN/dS*) in the Tephritidae, and investigated radical and conservative changes in amino acid physicochemical properties. We show a general selective constraint on *dsx*, but with signs of positive selection mainly in the common region. Such changes were localized in alpha-helices previously reported to be involved in dimer formation in the OD2 domain and near the C-terminal of the OD1 domain. In the population-based approach, we amplified a region of 540 bp that spanned almost all of the region common to both sexes from 32 different sites in Brazil. We investigated patterns of selection using neutrality tests based on the frequency spectrum and locations of synonymous and nonsynonymous mutations in a haplotype network. As in the divergence-based approach, these analyses showed that *dsx* has evolved under an overall selective constraint, but with some events of positive selection. In contrast to previous studies, our analyses indicate that even though *dsx* has indeed evolved as a conserved gene, the common region of *dsx* has also experienced bouts of positive selection, perhaps driven by sexual selection, during its evolution.

## Introduction

Even though several genes involved with premeiotic aspects of reproduction are exceptionally conserved throughout evolution [1], many genes related to reproduction in animals and plants are among the most divergent, evolving faster than those not related to sexual traits [2], possibly because of sexual selection and/or sexual conflict [3], [4]. In mammals, for example, many proteins related to fertilization are rapidly evolving [5]. In the same way, self-incompatibility genes in plants [6], gamete-recognition proteins from marine gastropods [7] and male and female reproductive proteins from *Drosophila* [8], [9] have shown patterns of rapid diversification. Most molecular studies so far have concentrated on genes involved in fertilization or male-female interaction and only a few on genes responsible for sexual differentiation. The gene *transformer* (*tra*), one of the central genes in the sexual differentiation cascade in Diptera [10], [11], *fruitless* from *Anastrepha* [12], and the *complementary sex determiner* gene (*csd*) from the honey bee cascade [13] are some of the examples of sex-determining genes whose molecular evolution have been studied.

The gene *doublesex* (*dsx*) plays a central role in the sex determination cascade. DSX is a transcription factor that acts by activating or repressing genes downstream in the cascade that are responsible for the development of male or female traits in insects [14]. This gene is functionally conserved among insects and other animals and, differently from other genes involved with sex determination [15]–[17], it occupies a conserved position at the bottom of the cascade [18], [19]. Notwithstanding its functional conservation, *dsx* varies both in its genomic organization and splicing pattern among insects [15], [20], [21]. In *Anastrepha*, *dsx* is composed of four exons: the first two are common to both sexes, the third exon is specifically female-expressed and the fourth is male-specific [22]. The common region contains a DNA-binding/dimerization domain (DM/OD1) at exon 1 and a second dimerization domain (OD2) at exon 2. Additionally, the OD2 spans the 5′-end of both female and male exons [22].The DNA-binding domain was described as a novel class of zinc-finger DM motif, which is structurally conserved among metazoans [14] and contains another oligomerization domain (OD1). Because of functional and structural conservation of the DM/OD1 motif and the central role *dsx* plays in sexual differentiation, this gene is expected to have a direct influence on fitness and should be, in general, reasonably conserved. On the other hand, *dsx* may be subject to sexual selection since it is also involved in many aspects of reproduction and sexual behavior [23] and, as a consequence, could show signs of positive selection.

Some studies performed in insects have shown a pattern of evolution for *dsx* which is compatible with the conservation of its role in sex determination across insects [18], [19], [24]. More recently, an analysis of the evolution of *dsx* in species of the genus *Anastrepha* suggested that this gene evolved primarily under purifying selection [25]. However, global estimates of evolutionary rates in coding regions have low power to detect signals of positively selected changes [26], and it is possible that even though the gene as a whole shows signs of purifying selection, different parts of the gene may behave differently. So, we investigated patterns of molecular adaptation of *dsx* in the *Anastrepha fraterculus* species group by testing the hypothesis that this gene is evolutionarily conserved and evolved solely under purifying selection. To do so, we used different approaches to assess long-term variation in nonsynonymous to synonymous rate ratio (*dN/dS*), amino acid property changes, and approaches based on population data used to study *dsx* evolution. Contrary to other studies [25], [27], our results indicate that even though *dsx* has indeed evolved as a conserved gene, some gene regions have experienced bouts of positive selection.

## Materials and Methods

Analysis of selection on *dsx* was performed with a hierarchical strategy. Initially, we evaluated evolutionary patterns using divergence-based methods with sequences from different species of *Anastrepha* and other Muscomorpha available on GenBank. In this context, we analyzed the data considering variation in nonsynonymous/synonymous ratios (*dN/dS* = *ω*) and changes in amino acid properties. We also wanted to test whether the same selective pattern would be detected at the population level. Thus, we sequenced a portion of the gene *dsx* in several individuals of *Anastrepha* from the *fraterculus* species group and performed different neutrality tests based both on the site frequency spectrum and the haplotype network topology.

### Long-term *doublesex* evolution

For the interspecific evolutionary study, we used the following complete *dsx* nucleotide sequences available from GenBank: *Musca domestica* (female: AY461853.1; male: AY461854.1), *Drosophila ananassae* (female and male: CH902617.1), *D. sechellia* (female and male: CH480821.1), *D. melanogaster* (female and male: AE014297.2), *D. erecta* (female and male: CH954181.1), *D. virilis* (female and male: XM_002056562.1), *D. pseudoobscura* (female and male: XM_001358983.2), *D. persimilis* (female and male: CH479179.1), *Bactrocera dorsalis* (female: FJ176944.1; male: FJ185162.1), *B. oleae* (female: AJ547621.1; male: AJ547622.1), *B. tryoni* (female: AF029675.1; male: AF029676.1), *B. correcta* (female: FJ185166.1; male: FJ185165.1), *Ceratitis capitata* (female: AF435087.2; male: AF434935.2), *Anastrepha obliqua* (female: AY948420.1; male: AY948421.1), *A. fraterculus sp1* (female: DQ494344.1; male: DQ494334.1), *A. fraterculus sp2* (female: DQ494325.1; male: DQ494335.1), *A. fraterculus sp3* (female: DQ494326.1; male: DQ494336.1), *A. fraterculus sp4* (female: DQ494327.1; male: DQ494343.1), *A. bistrigata* (female: DQ494332.1; male: DQ494341.1), *A. grandis* (female: DQ494328.1; male: DQ494337.1), *A. serpentina* (female: DQ494329.1; male: DQ494338.1), *A. sororcula* (female: DQ494330.1; male: DQ494339.1) and *A. striata* (female: DQ494331.1; male: DQ494340.1). Before phylogenetic reconstruction, we converted nucleotide sequences to amino acids and aligned them using *Clustal W* implemented in *BioEdit Sequence Alignment Editor* software [28] (Material S1 and S2). We used the amino acid alignment to set up the nucleotide alignment and perform subsequent evolutionary analyses.

Because *dsx* undergoes sex-specific alternative splicing, the protein is composed of a common region and sex-specific domains. The common region contains two conserved domains, OD1/DM and OD2, responsible for DNA-binding and protein dimerization, respectively. Considering such a complex organization and the possibility that each part of the gene may have different selective histories, we performed each analysis of selection separately: for analyses considering variation in *ω* and radical changes in physicochemical properties, the subsets were organized into female and male isoforms. In the comparison of evolutionary rates among different segments of *dsx*, we established four different subsets each representing a specific contrast. First, we inferred a phylogenetic tree for each subset by maximum likelihood using *MEGA* ver. 5 [29] using the nucleotide substitution estimated in *ModelTest* [30] implemented in *HyPhy* [31]. Considering that saturation of synonymous substitutions could underestimate *dS* rates, which might lead to overestimated *ω* ratios, we checked for saturation in synonymous and nonsynonymous substitutions by plotting proportions of synonymous and nonsynonymous nucleotide differences per synonymous and nonsynonymous sites, *pS* and *pN* respectively, against sequence divergence for pairs of taxa. Sequence divergences, *pS* and *pN* were estimated using the modified Nei-Gojobori method, with transition/transversion bias equal 2, implemented in *MEGA* ver. 5 [29]. Because recombination interferes with phylogenetic inferences, we performed three different methods to detect recombination events: the *Maximum Chisquare* [32] was performed in *MaxChi*, *GENECONV* [33], and *RDP* was implemented in *RDP* version 3b14 [34]. A cutoff limit of *p*<0.05 was set for the three tests and, when applicable, a randomization of 1000 replicates was performed.

### Positive selection detection by divergence-based methods: changes in *dN/dS*

We performed the branch-site test [35], which infers positive selection considering *ω* variation in branches and sites, using CODEML, implemented in PAML ver. 4 [36]. A *ω*>1 indicates positive selection because nonsynonymous substitutions have higher fixation probabilities than synonymous mutations due to selective advantages. On the other hand, a *ω*<1 indicates purifying selection caused by selective constraints at codons. For this test, a phylogenetic tree was separated in a *foreground* branch composed of the lineage of interest, and in *background* branches, represented by the other lineages [35]. In so doing, these tests evaluate whether patterns of selection in the foreground branches are significantly distinct from the patterns of the background branches, which would indicate that different patterns of selection have affected specific evolutionary lineages [37].

Positive selection was inferred by the contrast of distinct models using a maximum likelihood approach. We used an alternative model (MA), which constrains the codons in the *background* branches to 0<*ω*<1 or *ω* = 1 (*ω*_0_ and *ω*_1_ site classes, respectively) and allows the codons in the *foreground* to have *ω*>1 (*ω*_2_ site class) in addition to the other *ω*_0_ and *ω*_1_ site classes. The null model (MA_null_) sets up the same *ω* site classes of the alternative model MA, except that the *ω*_2_ site class in the *foreground* is fixed to 1. Positive selection was inferred with a significant log likelihood ratio test (LRT) of the alternative versus the null model. We used a chi-squared null distribution with 1 degree of freedom to assess significance of the LRT.

The signature of positive selection in individual codons was estimated by Bayesian analysis, in which *ω* was allowed to vary among sites. The *Bayes Empirical Bayes* method [37] was used in conjunction to the branch-site test to estimate which sites were under the influence of positive selection. Because some models are prone to problems of convergence in a likelihood framework, we ran the analyses twice with different initial *ω* values. These tests were performed in the subsets described above.

### Contrasting evolutionary rates among different regions in *dsx*

In order to compare the evolutionary rates among different segments of *dsx*, we established several different contrasts. For every contrast, we partitioned the molecule in two different regions to compare their evolutionary rates. We initially contrasted the common region to the male-specific and female-specific exons. We further contrasted the male-specific to the female-specific exon. Finally, we contrasted the evolutionary rates of the DM/OD1 and OD2 domains to the rest of the common region, which we defined as *variable common region*. For all data sets we tested the heterogeneity in evolutionary rates among each partition with the fixed-sites models implemented in PAML [38].

Variation in site heterogeneity was first assessed by contrasting the null model A to the alternative model B to test whether the regions had the same substitution rates. In model A, both partitions were treated as a single data set, with equal substitution patterns, equilibrium codon frequencies (*π*), transition/transversion rate ratio (*κ*), and nonsynonymous to synonymous rate ratio (*ω*). Model B separated each partition and estimated proportional branch lengths for each one, but estimated the same *π*, *κ* and *ω* for both partitions. This was followed by contrasting null model C versus alternative model E. Model C allows the partitions to have different branch lengths and π, but estimates the same *κ* and *ω* for both partitions, while model E allows each partition to have independent substitution rates. Then, the contrast between model C *vs* E tests for differences in *κ* and *ω* between partitions.

Models were contrasted using likelihood ratio tests (LRT) in which the degrees of freedom in each contrast were obtained by the difference in the number of parameters (1 and 2 degrees of freedom for the contrasts A *vs* B and C *vs* E, respectively). Because in the fixed-sites models *ω* is simultaneously estimated with *κ*, we cannot unambiguously determine if a significant LRT in the contrasts of models C *vs* E was due to *κ*, *ω* or both. Then, independent *κ* and *ω* values for these two regions obtained in model E were compared to determine if both parameters substantially differed between partitions.

### Positive selection detection by divergence-based methods: changes in physicochemical properties

We also analyzed the evolution of *dsx* sequences by studying changes in amino acid properties through evolutionary time with the *MM01* method [39] which considers changes in many physicochemical properties caused by each nonsynonymous substitution. We carried out these analyses in *TreeSAAP* ver 3.2 [40] separately for the complete male and female isoforms using each respective maximum likelihood phylogenetic tree. In this analysis, global deviation from neutrality was initially verified by a goodness-of-fit test between a neutral expected distribution and the observed distribution of changes in selected physicochemical properties. The magnitude of nonsynonymous changes was then classified into three categories according to the change in specific physicochemical properties, from conservative (category 1) to very radical substitutions (category 3). For each category, a z-score was calculated, which was compared with a mean among all categories. Significant positive z-scores showed that the number of inferred amino acid replacements significantly exceeds the number expected by chance, thus indicating that the region is under the influence of positive selection. Significant negative z-scores indicated a signal of purifying selection since the inferred nonsynonymous changes were significantly less frequent than expected by chance. When a significant positive z-score was detected in the more conservative category 1, the property was considered to be under stabilizing selection. Conversely, the property was considered to be under destabilizing selection when a significant positive z-score was detected in the more radical category 3. McClellan et al. [39] defined stabilizing selection that tends to maintain the original biochemical attributes of the protein despite the inference of positive selection, and destabilizing selection as that which favors structural and functional shifts in a region of a protein. In this way, positive-destabilizing selection represents a signature of molecular adaptation.

We analyzed 51 amino acid physicochemical properties and considered only 3 categories of magnitude changes despite the 8 categories originally defined [39] to avoid false positives, as suggested by McClellan and Ellison [41]. Only amino acid properties identified by significant positive z-scores in category 3 were considered to be affected by positive-destabilizing selection. To verify which specific regions were affected by positive-destabilizing selection, we performed a sliding window analysis using only the amino acid properties that were significant for positive-destabilizing selection. Sliding windows of 20 codons with a sliding step of one codon were selected for showing the best accuracy [41], [42].

### Diversity and population level analysis of *doublesex*

**Figure 1:**
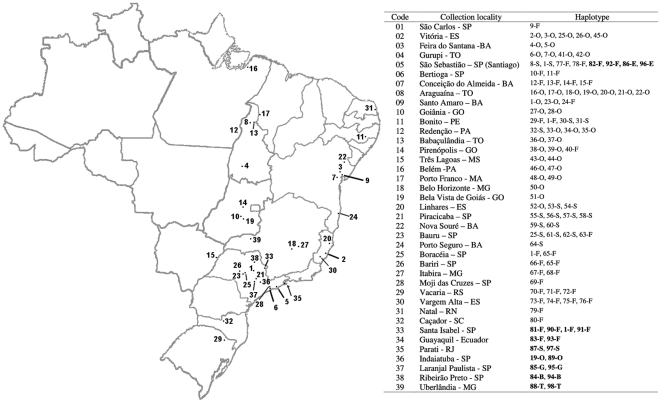
Map of sampling sites in Brazil. The haplotypes from each site are shown in the table at right.

We sampled 51 individuals of *A. fraterculus*, *A. obliqua* and *A. sororcula* from 32 localities in Brazil (Figure 1 and Table S1). DNA extraction was performed using a modified procedure of Nelson and Krawetz [43] to ensure that the exoskeleton remained intact for future morphological analyses. We amplified a region of 540 bp that spanned almost all of the exon common to both sexes (bases 34 to 574) and contains the DM/OD1 domain using degenerate primers created from homologous sequences of closely related species (5′– ATGGTTTCNGAGGATAATTG –3′ and 5′– GCGNCCNACNACYGANATNGGCAA –3′). This region was PCR amplified from genomic DNA in a BioRad PTC-200 thermocycler using a mixture of *Taq* polymerase and *Pfu* polymerase to reduce incorporating errors [44]. PCR products were purified by PEG 8000 precipitation [45] and cloned using the InsTAclone kit (Fermentas). At least two recombinant colonies were sequenced per individual with forward and reverse M13 primers using the DYEnamic™ ET dye terminator kit (GE Healthcare) and resolved in a MegaBace 1000 (GE Healthcare). Quality of base-calling was visually inspected in Chromas version 2.31 (http://www.technelysium.com.au). Because these sequences were very similar, they were aligned and visually inspected using *BioEdit Sequence Alignment Editor* [28]. When equal or very similar sequences differing by 1–2 bases were obtained from clones of the same individual, additional sequencing was performed on different recombinant colonies (up to five total) to confirm eventual homozygosity of individuals or sequencing errors. All sequences were deposited in GenBank (Table S2). Because recombination interferes with phylogenetic inferences, we performed the same tests for recombination used in the divergence-based analyses.

Population level analyses were performed on this 540 bp fragment from the common region. We estimated haplotype (*Hd*) and nucleotide diversity (*π*) [46], number of polymorphic sites, synonymous nucleotide diversity (*π*_s_), nonsynonymous nucleotide diversity (*π*_a_) and Watterson's *θ* using *DnaSP* ver. 4.10.9 [47]. Tajima's *D* [48], Fu and Li's *D* and *F* [49] and Fay and Wu's *H* [50] neutrality tests were performed in *DnaSP* ver. 4.10.9 [47] using *Bactrocera dorsalis* sequence (GenBank accession number AY669318) as an outgroup. Similar to the analysis of the long-term dataset, we separated this region in a conserved common subset, composed only of the DM/OD1 domain and a variable common subset, composed of the common region without the DM/OD1 domain. This subdivision was also used in further selection tests based on haplotype networks. Therefore, diversity indexes and neutrality tests were estimated for these two subsets separately, as well as for them combined (the whole region amplified). The significance levels of all neutrality tests and comparisons of diversity indexes were established by the use of a Dunn-Šidàk correction [51] for multiple test with the Holm's step-down algorithm [52].

### Selection tests based on an intraspecific haplotype network

Departures from neutrality were also tested using the distribution of synonymous and nonsynonymous substitutions in tip and interior haplotypes (young and old haplotypes, respectively) in a haplotype network using Fisher's Exact Test on a 2×2 contingency table [53]. This is a more refined version of the McDonald and Kreitman's test (MKA) [54] used to study intra and interspecific polymorphisms. The haplotype network used in this test was inferred by statistical parsimony using *TCS* 1.21 [55]. We expected under neutrality that the ratio of nonsynonymous to synonymous mutations in tip haplotypes (younger haplotypes) should be the same as the ratio of nonsynonymous to synonymous mutations in interior haplotypes (older haplotypes). Under purifying selection, the nonsynonymous to synonymous ratio is expected to be lower in interior than in tip haplotypes. On the other hand, when recurrent events of directional positive selection or balancing selection occur on the gene, we expect the nonsynonymous to synonymous ratio to be greater in interior than in tip haplotypes.

We also performed this test contrasting the fate of synonymous and nonsynonymous mutations in conserved (DM/OD1) and variable segments of the common region. The complete test considered synonymous and nonsynonymous mutations in tip and interior haplotypes. In this analysis, the null hypothesis of homogeneity of data was simulated by 1000 random permutations and the test of hypothesis was conducted with an exact permutation test using the algorithm of Roff and Bentzen [56].

We used an additional test for selection which evaluated whether synonymous substitutions have a greater or smaller probability of surviving in the population when compared to nonsynonymous substitutions [12]. In this test, the survival of a mutation is evaluated by the frequency of haplotypes derived from it. The older the haplotype, the greater the probability it will be more frequent [57] and the greater the chance to be internal in an intraspecific haplotype network [58]. It is, then, reasonable to apply these same expectations to the mutations that gave rise to these haplotypes. In this way, we ranked the number of haplotypes in the network derived from internal synonymous and nonsynonymous mutations, and compared the sum of the ranks through an improved normal approximation to Mann-Whitney's U test [59] to establish a comparison between their survival probabilities.

In a neutrally evolving region, we expected the same survival probability through time for synonymous and nonsynonymous mutations, so we did not expect to see a significant difference in their ranks. On the other hand, if the region is under purifying selection, nonsynonymous mutations would have a higher chance of being eliminated and synonymous of being maintained. Therefore, in this case we expected that synonymous mutations would be on average older, and as a consequence would be more frequent, and then, would have significant higher ranks than nonsynonymous mutations. The opposite would be expected if balancing selection or recurrent positive directional selection have occurred on the selected region [60]–[62]. Because recent mutations, such as those in the tips of the network and with frequency of one or two derived haplotypes, may not yet have passed through the evolutionary test of survival and reproduction over time [53], and may be much more affected by drift, we only considered mutations present in at least three haplotypes.

## Results

### Long-term evolutionary analysis of *doublesex*

**Figure 2:**
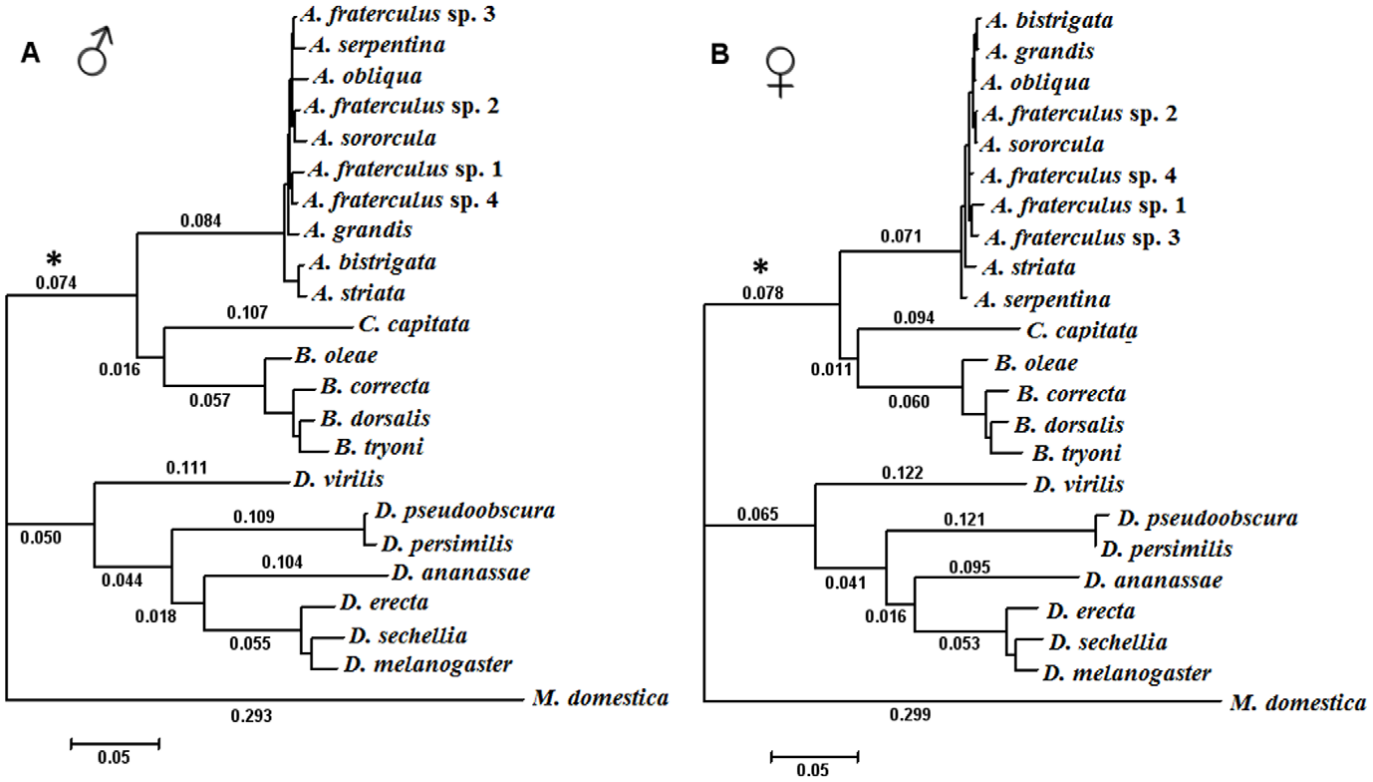
Unrooted phylogenetic trees estimated by maximum likelihood. A) Male isoform. B) Female isoform. The *foreground* branch is marked by an asterisk (*). The branch lengths are as nucleotide substitutions per nucleotide site.

**Figure 3:**
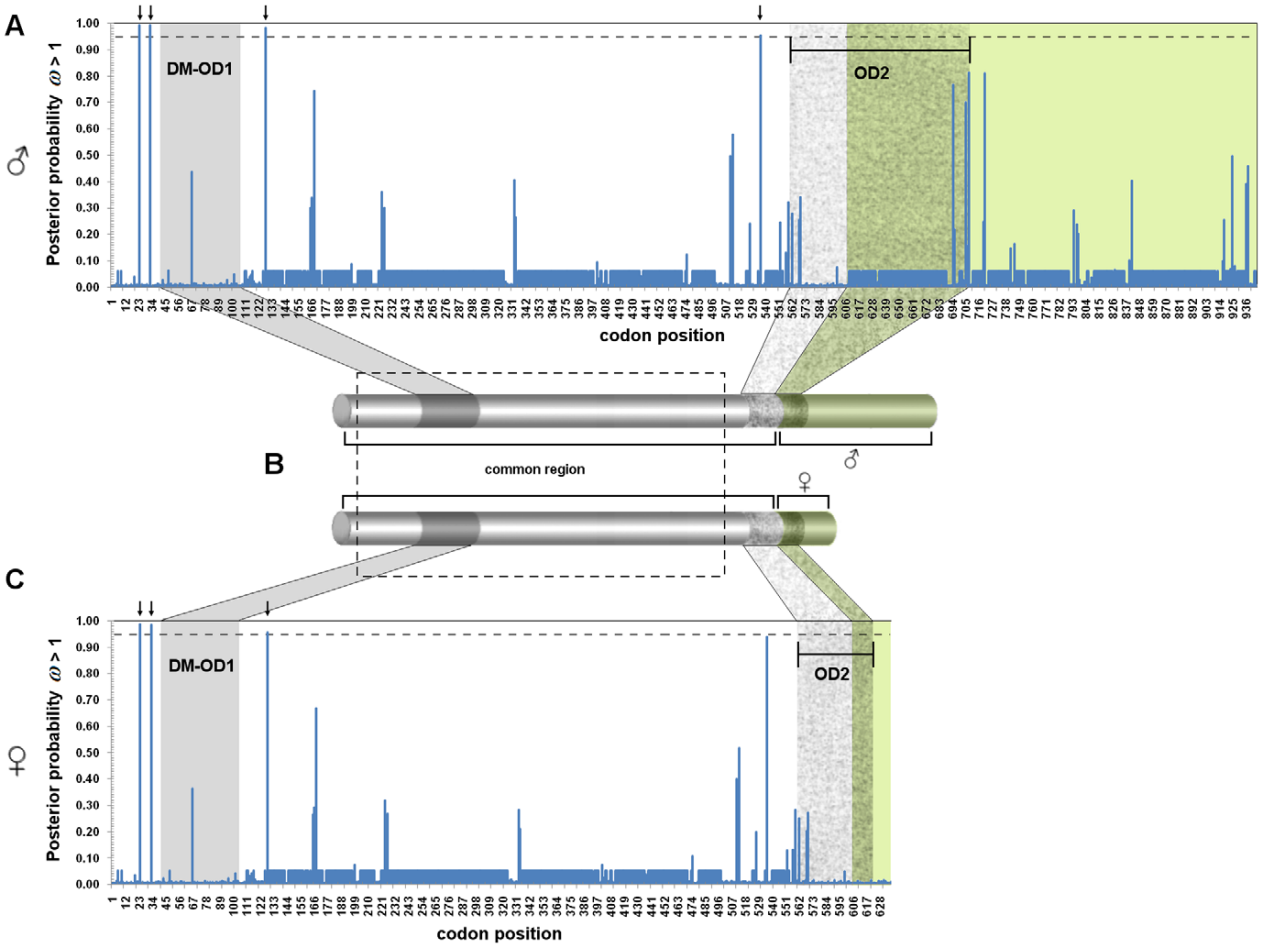
*Bayes Empirical Bayes* of male and female isoform. A) Posterior probability for *ω* >1 in male isoform. B) Schematic representation of male and female transcripts. The dashed box stands for the amplified segment used in populational analyses. C) Posterior probability for *ω*>1 in female isoform. The dashed line represents the 0.95 limit of posterior probability for *ω*>1. Arrows indicate codon positions with posterior probability greater than 0.95 for *ω* >1. DM/OD1 – DNA-binding and dimerization domain 1. OD2 – dimerization domain 2.

Contrasts between the MA and MA null models of branch-site test considering the male and female isoforms, performed in the framework of the inferred ML phylogenetic trees depicted in Figure 2, confirmed that part of the differentiation that occurred in *dsx* was due to positive selection (Table 1). The signals of positive selection were particularly evident in sites outside the DM/OD1 and OD2 domains, as indicated by the Bayes Empirical Bayes analyses (Figure 3). Because we are contrasting well-diverged lineages, the inferences of *ω* would be biased due to synonymous saturation. However, these inferences based on elevated *ω* rates are warranted, since these contrasts are performed among lineages that show at most 0.07 substitutions per nucleotide site, which is lower than the point in which we began detecting significant synonymous saturation (0.25 substitutions per nucleotide site) (Figure S1).

**Table 1:** Parameters estimates and log-likelihood for models MA and MA null and contrast between models MA and MA null by likelihood ratio test (LRT).

Data set	Model	Parameters estimates	log-likelihood	LRT	Positively selected sites
Male isoform	MA	*ω*_0_ = 0.068, *ω*_1_ = 1.000, *ω*_2_ = 16.906, p_0_ = 0.885, p_1_ = 0.073, (p_2_+p_3_) = 0.042	−10154.136	8.872**	**24, 33, 128, 536**
	MA null	*ω*_0_ = 0.067, *ω*_1_ = 1.000, *ω*_2_ = 1.000, p_0_ = 0.841, p_1_ = 0.071, (p_2_+p_3_) = 0.007	−10158.572		Not allowed
Female isoform	MA	*ω*_0_ = 0.059, *ω*_1_ = 1.000, *ω*_2_ = 11.786, p_0_ = 0.901, p_1_ = 0.077, (p_2_+p_3_) = 0.023	−7307.287	6.149**	**24, 33, 128**
	MA null	*ω*_0_ = 0.058, *ω*_1_ = 1.000, *ω*_2_ = 1.000, p_0_ = 0.881, p_1_ = 0.075, (p_2_+p_3_) = 0.044	−7310.361		Not allowed

*ω*_0_ = *dN/dS* for sites with 0<*ω*<1 ; *ω*_1_ = *dN/dS* for sites with *ω* = 1; *ω*_2_ = *dN/dS* for sites with *ω*>1. p_0_ = proportion of sites with *ω*_0_; p_1_ = proportion of sites with *ω*_1_; (p_2_+p_3_) = proportion of sites with *ω*_2_; All such parameters correspond only to *foreground* branches. The LRT critical values at 5% and 1% level are 2.71(*) and 5.41(**), respectively. Site numbers in boldface are at the common region.

### Contrast between evolutionary rates in different segments of *dsx*

Even though the contrast of model A *vs* B failed to show significant differences in their substitution rates when we compared the common region to the male-specific exon, models B and C were rejected in the contrasts B *vs* D and C *vs* E, respectively (Table 2), which may have been caused by significant differences in *κ* and/or *ω*. Because *ω* was practically the same between the two regions, whereas *κ* was clearly the most divergent, the significance of the contrasts was probably due to a difference in the rates of transitions to transversions between male-specific and common exons.

**Table 2:** Contrasts of evolutionary rates between different regions of *doublesex* by the fixed-sites test.

Contrasted regions	Model contrast	LRT	D.F.	Probability (χ^2^)	Relative substitution rates	*κ* estimates under model E and D	*ω* estimates under model E and D
common region *vs* male exon	A×B	0.819	1	0.365	r_common_ = 1.0	*κ*_common_ = 1.75	*ω*_common_ = 0.09
	B×D	**7.963**	2	**0.019**	r_male_ = 0.9	*κ*_male_ = 2.45	*ω*_male_ = 0.11
	C×E	**11.931**	2	**0.003**			
common region *vs* female exon	A×B	**65.334**	1	**6.322 10^−16^**	r_common_ = 1.0	*κ*_common_ = 1.62	*ω*_common_ = 0.09
	B×D	4.296	2	0.117	r_female_ = 0.3	*κ*_female_ = 3.47	*ω*_female_ = 0.08
	C×E	2.521	2	0.284			
female exon *vs* male exon	A×B	**56.14**	1	**6.665 10^−14^**	r_female_ = 1.0	*κ*_female_ = 3.73	*ω*_female_ = 0.07
	B×D	1.808	2	0.405	r_male_ = 3.4	*κ*_male_ = 2.58	*ω*_male_ = 0.10
	C×E	1.220	2	0.543			
DM/OD1 vs variable common region	A×B	**9.26**	1	**0.002**	r_DM/OD1_ = 1.0	*κ*_DM/OD1_ = 2.17	*ω*_DM/OD1_ = 0.01
	B×D	**78.17**	2	**1.061 10^−17^**	r_common_ = 1.3	*κ*_common_ = 1.77	*ω*_common_ = 0.12
	C×E	**77.11**	2	**1.803 10^−17^**			
OD2 vs variable common region	A×B	**63.49**	1	**1.612 10^−15^**	r_OD2_ = 1.0	*κ*_OD2_ = 2.19	*ω*_OD2_ = 0.06
	B×D	**30.45**	2	**2.442 10^−7^**	r_common_ = 2.05	*κ*_common_ = 1.85	*ω*_common_ = 0.13
	C×E	**22.88**	2	**1.077 10^−15^**			

LRT – Likelihood Ratio Test; D.F. – degrees of freedom. Significant contrasts are in boldface.

When we compared the female exon to the common region, only the contrast of models A *vs* B was significant, showing a 3-times smaller substitution rate for the female exon, suggesting a stronger selective restriction on the female exon. Because the contrasts of models B *vs* D and C *vs* E were not significant, there was little evidence that the difference in rates between common region and female exon were due to differences in *κ* and/or *ω* rates. Similarly, the female exon showed a 3.4 times reduction in substitution rate in relation to the male exon, but with little evidence for significant differences in *κ* and/or *ω* rates.

### Positive selection detection by changes in physicochemical properties

When we considered the effects of selection on physicochemical changes in the male and female isoforms, 27 and 29 out of 51 properties, for female and male respectively, deviated significantly from neutrality, based on the global goodness-of-fit statistics calculated by the *MM01* method in TreeSAAP (Table S3). The significant properties were examined by a more specific analysis which separated nonsynonymous changes into three categories and tested if each category significantly deviates from neutrality. We found three amino acid properties to be under positive-destabilizing selection (category 3) in the male isoform; among them, two amino acid properties were related to changes in alpha-helix structure and one was related to changes in beta-sheet structure (Table 3). However, only the *Normalized Frequency of Alpha-helix* property was under positive-destabilizing selection in the female isoform (Table 3). We found 16 and 18 amino acid properties under positive-stabilizing selection (purifying selection) in female and male isoforms. In the male isoform, six properties were involved in hydrophobic interactions, six in charge/polarity changes, two in beta-sheet structure and four in turn/bend structural changes. In the female isoform, six physicochemical properties were related to hydrophobic interactions, seven to charge/polarity changes, and three to bend/beta-sheet structures (Table 3).

**Table 3:** Amino acid physicochemical properties under positive destabilizing and purifying selection in male and female isoforms of *doublesex*.

Property	Goodness-of-fit neutral expectation	z-score in conservative change (category 1)	z-score in radical change (category 3)
**Male isoform**			
Alpha-helix propensity derived from designed sequences (KOEP990101)	7.004*	-	2.354**
Average relative probability of beta-sheet (KANM800102)	13.771**	-	1.916*
Beta-sheet propensity derived from designed sequences (KOEP990102)	27.322***	3.100***	-
Beta-strand indices for beta-proteins (GEIM800106)	31.892***	2.141*	-
Hydrophilicity value (HOPT810101)	102.508***	4.165***	-
Hydrophobic parameter (LEVM760101)	101.53***	4.165***	-
Hydrophobicity (JOND750101)	23.206***	2.346**	-
Hydrophobicity (PRAM900101)	50.67***	2.556**	-
Hydrophobicity factor (GOLD730101)	25.074***	2.475**	-
Hydrophobicity index (ARGP820101)	23.206***	2.346**	-
Isoelectric point (ZIMJ680104)	98.935***	4.061***	-
Net charge (KLEP840101)	96.412***	3.890***	**-**
Normalized frequency of alpha-helix (BURA740101)	8.490*	-	2.350**
Normalized frequency of reverse turn, with weights (LEVM780103)	13.843***	1.906*	**-**
Normalized frequency of turn from LG (PALJ810105)	9.347**	1.811*	**-**
Normalized relative frequency of bend (ISOY800103)	17.201***	2.199*	**-**
Normalized relative frequency of double bend (ISOY800107)	29.789***	2.363**	**-**
pK (-COOH) (JOND750102)	35.418***	1.933*	**-**
Polarity (GRAR740102)	55.985***	3.251***	**-**
Polarity (ZIMJ680103)	52.857***	3.244***	**-**
Positive charge (FAUJ880111)	29.904***	2.179*	**-**
**Female isoform**			
Beta-sheet propensity derived from designed sequences (KOEP990102)	26.713***	2.974**	-
Beta-strand indices for beta-proteins (GEIM800106)	27.528***	1.990*	-
Hydrophilicity value (HOPT810101)	93.536***	4.094***	-
Hydrophobic parameter (LEVM760101)	92.808***	4.094***	-
Hydrophobicity (JOND750101)	14.690***	1.858*	-
Hydrophobicity (PRAM900101)	40.302***	2.406**	-
Hydrophobicity factor (GOLD730101)	17.573***	2.062*	-
Hydrophobicity index (ARGP820101)	14.690***	1.858*	-
Isoelectric point (ZIMJ680104)	89.787***	3.998***	-
Negative charge (FAUJ880112)	39.901***	1.679*	-
Net charge (KLEP840101)	86.841***	3.804***	-
Normalized frequency of alpha-helix (BURA740101)	6.760*	-	2.249*
Normalized relative frequency of double bend (ISOY800107)	30.651***	2.460**	-
pK (-COOH) (JOND750102)	29.895***	1.827*	-
Polarity (GRAR740102)	45.076***	2.941**	-
Polarity (ZIMJ680103)	45.213***	3.107***	-
Positive charge (FAUJ880111)	20.636***	1.868*	-

Properties under positive destabilizing selection are boldfaced.

* - *p*<0.05;

** - *p*<0.01;

*** - *p*<0.001.

**Figure 4:**
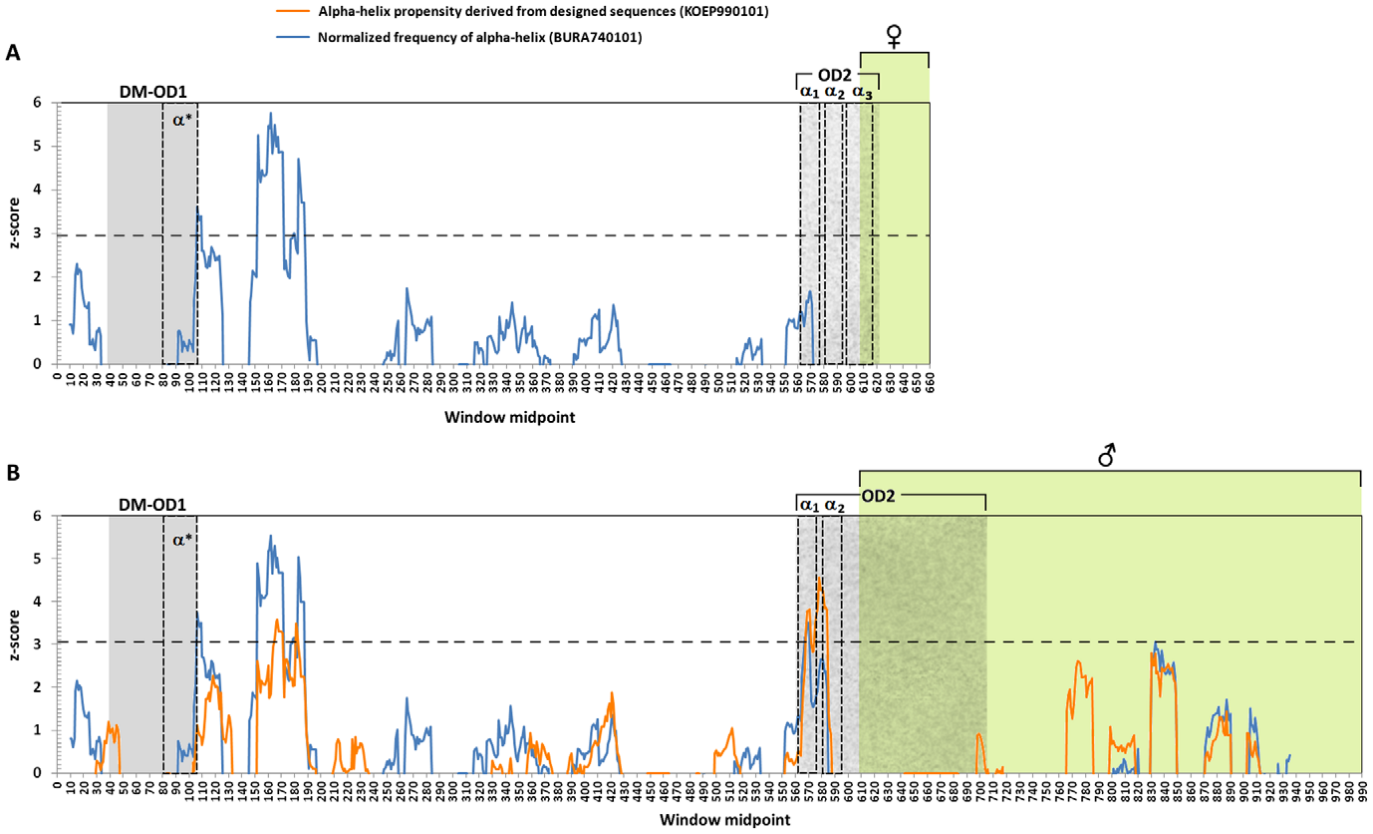
Sliding window plots of the z-scores of radically changed properties showing regions under positive-destabilizing selection in (A) female and (B) male isoform. DM/OD1 – DNA-binding and dimerization domain. OD2 – dimerization domain 2. α_1_, α_2_ and α_3_ – alpha-helices that compose the predicted UBA-like domain. α* - disordered C-terminal tail, proposed to fold as an alpha-helix. Dashed horizontal line indicates the Bonferroni corrected significant limit (z-score = 3.07, *p*<0.05, male exon; z-score = 2.95, *p*<0.05, female exon).

When the male and female isoforms were screened by the sliding windows approach, the majority of codons under positive-destabilizing selection were found in the common exons, particularly in a region near the 3′- end of DM/OD1 and in the male-specific OD2 domains (Figure 4). Among the properties previously detected to be under positive-destabilizing selection, only those related to modifications in alpha-helices were significant in the sliding windows approach. Likewise, only in the male isoform could we identify positive-destabilizing selection in the OD2 domain, specifically inside regions predicted to form alpha-helices of UBA-like domain responsible for the dimerization of DSX [63], [64].

### Population evolutionary analysis of *doublesex*

**Figure 5:**
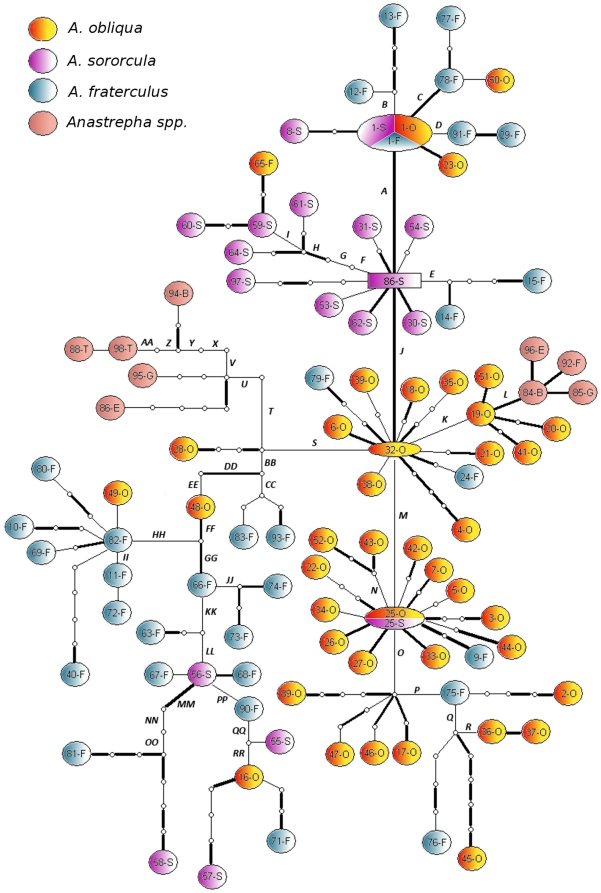
Haplotype network estimated by statistical parsimony (TCS ver 1.21). Thicker lines represent nonsynonymous substitutions and small circles stand for inferred haplotypes. Haplotypes were labeled sequentially and the subsequent letters represent the respective identified species. O – *A. obliqua*, F – *A. fraterculus*, S – *A. sororcula*, G – *A. grandis*, T – *A. striata*, B – *A. bistrigata*, E– *A. serpentina*. Letters near lines represent internal mutations labels.

The 540 bp amplified region of *doublesex* (Figure 3B) was cloned and sequenced from 51 individuals across Brazil (Figure 1) and combined with other sequences available from GenBank [25] for analysis. There were 98 different haplotypes, the great majority of which were singletons. Only six haplotypes were found in more than one copy, three of them shared among different species, including the most common, haplotype 1, which was found in *A. fraterculus*, *A. obliqua* and *A. sororcula* (Table S4). The different recombination tests failed to detect recombination in these sequences. The haplotype network inferred in TCS revealed that most variation did not define species-specific lineages (Figure 5). Because we failed to detect reciprocal monophyly, many of the parameters were also estimated for the group of species as a whole, considering the clade as the evolutionary unity of interest. For the same reason, MKA tests were not performed because it requires at least some fixed interspecific differences to compare intra and interspecific synonymous to nonsynonymous rate ratios.

Estimates of nucleotide and haplotype diversities, and nonsynonymous and synonymous substitutions were similar regardless of whether *A. fraterculus*, *A. obliqua* and *A. sororcula* were considered separately or as a single group along with other species of the *fraterculus* group (Table 4). We also failed to observe significant differences in *Hd*, *π* and *θ* between the DM/OD1 domain and the remaining sites of the common region.

**Table 4:** *doublesex* genetic diversity estimates of the common region and DM/OD1 domain.

Domain/segment	species	N	h	Hd (SD)	*π* (SD)	*π*_s_	*π*_a_	*θ* (SD)
full common region	*Anastrepha* sp.	109	97	0.995 (0.003)	0.016 (0.001)	0.037	0.009	0.056 (0.014)
	*A. fraterculus*	39	35	0.993 (0.008)	0.018 (0.001)	0.039	0.010	0.035 (0.011)
	*A. obliqua*	44	40	0.995 (0.007)	0.011 (0.001)	0.022	0.007	0.033 (0.010)
	*A. sororcula*	18	18	1.000 (0.019)	0.016 (0.003)	0.038	0.009	0.025 (0.009)
DM/OD1	*Anastrepha* sp.	109	58	0.936 (0.016)	0.015 (0.001)	0.032	0.010	0.054 (0.015)
	*A. fraterculus*	39	24	0.955 (0.018)	0.017 (0.002)	0.037	0.012	0.031 (0.011)
	*A. obliqua*	44	23	0.813 (0.060)	0.008 (0.002)	0.011	0.007	0.029 (0.010)
	*A. sororcula*	18	13	0.928 (0.052)	0.018 (0.004)	0.044	0.010	0.028 (0.011)
common region without DM/OD1	*Anastrepha* sp.	109	79	0.986 (0.005)	0.018 (0.001)	0.042	0.010	0.056 (0.014)
	*A. fraterculus*	39	32	0.988 (0.009)	0.018 (0.001)	0.041	0.011	0.035 (0.011)
	*A. obliqua*	44	36	0.985 (0.010)	0.014 (0.001)	0.029	0.009	0.033 (0.010)
	*A. sororcula*	18	14	0.954 (0.039)	0.017 (0.003)	0.034	0.011	0.025 (0.010)

N = number of sequences; h = number of haplotypes; Hd = haplotype diversity; S = number of polymorphic sites; Sy = number of synonymous changes; Nsy = number of nonsynonymous changes; *π* = nucleotide diversity; *π*s = synonymous nucleotide diversity; *π*a = nonsynonymous nucleotide diversity. SD = standard deviation.

Tajima's *D* and Fu and Li's *D* were significant only in *Anastrepha* sp. and *A. obliqua* (Table 5) in the direction of purifying selection. The same signal of purifying selection was found using Templeton's contingency test, which showed a significant excess of nonsynonymous substitutions in tips and synonymous substitutions in interior branches (Tables 6 and 7). Even when DM/OD1 was removed from the common region and both subsets were independently analyzed, we could still reject the null hypothesis of equality in proportions of synonymous and nonsynonymous mutations in tip and interior branches (Table 8).

**Table 5:** Neutrality tests for *dsx* DM/OD1 and common region without DM/OD1 and OD2 domains.

Domain/segment	Species	Tajima's *D*	Fu and Li's *D*	Fay and Wu's *H*
full common region	*Anastrepha* sp.	−**2.367** **	**−6.386** **	−34.402
	*A. fraterculus*	−1.782	−3.000^a^	−14.826
	*A. obliqua*	**−2.411** **	**−5.087** **	−7.904
	*A. sororcula*	−1.376	−2.050^a^	−3.719
DM/OD1	*Anastrepha* sp.	**−2.236** *	**−5.098** *	−9.657
	*A. fraterculus*	−1.475	**−2.367** *	−4.920
	*A. obliqua*	**−2.354** **	**−4.136** **	−3.824
	*A. sororcula*	−1.399	−1.665	−4.760
common region without DM/OD1	*Anastrepha* sp.	**−2.345** **	**−6.414** **	−17.489
	*A. fraterculus*	−1.849	**−2.908** *	−9.905
	*A. obliqua*	**−2.298** **	**−4.769** *	−4.080
	*A. sororcula*	−1.34039	−1.977	1.041

Significant tests after Dunn-Šidàk correction are given in bold.

** *p*<0.01;

* *p*<0.05; a 0.10>*p*>0.05.

**Table 6:** Contingency analysis of synonymous and nonsynonymous vs tip or interior position in the haplotype network.

Position in the network	synonymous	nonsynonymous
Tip	71	95
Interior	34	9

Fisher's Exact Test probability under the null hypothesis of homogeneity: 2.7 10^−6^.

**Table 7:** Permutation analysis of synonymous and nonsynonymous mutations versus position in the haplotype network (tip or interior).

	synonymous		nonsynonymous	
Position in the network	DM/OD1	common region	DM/OD1	common region
Tip	24	47	35	60
Interior	11	23	3	6

Permutational probability: 0.0004.

**Table 8:** Contingency analyses of how synonymous and nonsynonymous mutations within DM/OD1 and common region are distributed across the haplotype network position (tip or interior).

	DM/OD1	
Position in the network	synonymous	nonsynonymous
Tip	24	35
Interior	11	3

Fisher's Exact Test probability under the null hypothesis of homogeneity: 0.0162.common region		
	common region	
Position in the network	synonymous	nonsynonymous
Tip	47	60
Interior	23	6

Fisher's Exact Test probability under the null hypothesis of homogeneity: 0.0008.

We failed to find any significant differences in the number of haplotypes derived from synonymous and nonsynonymous mutations if the full fragment was considered or if only the DM/OD1 subset was analyzed using Mann-Whitney's U test (data not shown). However, an analysis of the variable common region detected a significant difference in survival through time between synonymous and nonsynonymous mutations as measured by the number of derived haplotypes (Figure 5, Table S5). Despite the smaller number of interior nonsynonymous mutations, they gave rise to a significantly greater number of derived haplotypes (median = 22) than synonymous mutations (median = 13), (*Z_c_* corrected for continuity = 1.933; *p* = 0.052).

## Discussion

### Positively selected sites in *dsx*

Considering that *dsx* is critical to the differentiation of insects into males and females, mutations in *dsx* that cause deleterious effects might seriously impair reproduction. Mutations should have stronger effects if they occur in functionally conserved regions, as it has been shown for mutations in either DM/OD1 domain or OD2 domain that produced intersex phenotypes [65]. So, we might expect a strong selective constraint in *dsx*, with a consequent reduction in nonsynonymous variation.

In agreement with this expectation, previous studies have shown that *dsx* has been evolutionarily conserved, and concluded that the whole gene has evolved under purifying selection and that positive selection had played a minor role in the evolution of *dsx* in insects [15], [25], [27], [66]. However, these studies based their conclusions either on the percentage of identity among *dsx* sequences of different species [15], [25], [27] or on the overall effect of selection on the gene [25], [66] and they indicated the female exon to be the most conserved exon, followed by the male and common exons [15], [25], [27]. Likewise, when we compared evolutionary rates of female, male and common exons with each other, using the fixed-sites tests, we observed a stronger selective constraint on the female exon. Nonetheless, we failed to observe significant differences in evolutionary rates between the male exon and the common region. The low values of *ω* point estimates for the three main segments of *dsx* suggested that, overall, they have evolved selectively constrained.

Analyses that use global estimates of evolutionary rates in coding regions, though, have low power to detect signals of positively selected changes, and any gene segment under strong purifying selection could dramatically reduce the global *ω* estimates, even if there were sites outside the selectively constrained region under positive selection [26], [38]. Therefore, when we employed analyses which considered variation in *ω* rates in branches and sites, not only average estimates for *ω*, we found that *dsx*, although still under some selective constraints, showed several positively selected sites in the common region. The signal of positive selection was particularly related to structural changes in alpha-helices, which play important role in the dimerization and interaction of DSX with other transcription factors [14], [63], [67]. These tests contrast somewhat distant evolutionary lineages, therefore, their power may be limited by homoplasy, which is revealed particularly if there is saturation of synonymous substitutions when compared to evolutionary distances. If we had significant saturation in our estimates, we would have to consider the results of the analyses based on the *dN/dS* ratio with caution. Since the lineages here investigated are in the range of estimates without evidence of underestimation of *dS*, the evidence of positive selection by elevated *ω* rates here detected should hold.

### Selection and genetic variation at the population level

We wanted to investigate if the positive selection detected by comparing well diverged lineages would affect variation in *dsx* at the population level and whether we could also detect any evidence of positive selection at this level. Different species in the *A. fraterculus* group revealed high levels of haplotype diversity (*Hd*) and an excess of unique and rare haplotypes, with only a handful of haplotypes that were found more than once. Our sampling scheme, which favored few individuals from different and distant localities, may partially explain the excess of rare haplotypes observed, but it does not account for the high proportion of heterozygotes observed. Therefore, it is possible that the level of polymorphism observed is representative of the whole population. In spite of this sampling scheme, the polymorphism levels detected are not associated with fixed species-specific differences, a result consistent with other studies in the *fraterculus* species group using mitochondrial COI and some nuclear genes [12], [22], [25], [68].

The Templeton's contingency test indicated that the majority of nonsynonymous substitutions were present in tip haplotypes, which generally represent newly arisen mutations [48], rather than in interior haplotypes, which are expected to be older under neutrality [69]. This result along with the significantly negative Tajima's *D*, and Fu and Li's *D* and *F* neutrality tests in *Anastrepha* sp. and *A. obliqua* is compatible with an overall signature of purifying selection. Such results concur with what was observed with the haplotype diversity and indicate an excess of rare variants that fit the expectations of background selection [70]. Though these neutrality tests are generally used to detect the overall effects of selection on a DNA region, similar results might also be obtained by some demographic factors, such as Wahlund effect or population size expansion, even in the absence of selection [71].

In spite of the evidence of purifying selection in the sequenced fragment of *dsx* from some neutrality tests, we observed several nonsynonymous substitutions that were very frequent in the populations, including some that were involved with radical changes in amino acid properties. The Mann-Whitney *U* test showed that even though internal nonsynonymous mutations are rarer, their descendents are present in higher number of copies in the population than the descendents of internal synonymous mutations. This pattern deviates significantly from the neutral expectation, and suggests the action of positive selection on the common region of *dsx* also at the population level, again in a framework of overall purifying selection.

### Selection patterns in *dsx*

While little is known about the mechanism by which positive selection could have occurred on *dsx* (but see [66]), the pattern of variation observed for the common region of *dsx* might be explained by the dual nature of DSX. On one hand, DSX is a central part of the conserved sex-determination cascade and also interacts with a variety of genes including those involved in homeotic pathways [72]–[74]. On the other, DSX is involved in several other reproductive functions including male courtship behavior, along with FRU [23], and genital development [72], [75], which may be subject to sexual and positive selection. Consequently, changes in DSX may be selectively disadvantageous for one function, but not for another. It is even possible that because of such complex interactions, mutations at *dsx* could behave as slightly deleterious or advantageous mutations depending on the genetic background they are interacting with, a process that resemble sign epistasis [76].

Another mechanism that could favor positive selection might be the interaction among different segments of DSX. DSX is functional as a homodimer, and the dimerization is carried out by DM/OD1 and OD2 domains [14], [77]. Both domains are thermodynamically linked, since the binding of DM to DNA is favored by the strengthening of the dimerization by OD2 domain [78]. Thus, important changes in the structure and/or chemical interaction in one domain might induce changes in the other, and might explain the positive-destabilizing selection for alpha-helices detected near the C-terminal of OD1 and inside the OD2.

Such pattern of selective constraint in important domains and diversification in other parts of the gene seems to be common for other sex-determining genes, such as *transformer* (*tra*) and *fruitless* (*fru*). The gene *tra* has been shown to be relatively unconstrained, with a *ω* rate ratio moderately higher (*ω* = 0.2 to 0.3) than that found for other genes not related to sex differentiation [10], [11]. These data indicate, nonetheless, that *tra* in *Drosophila* is still under purifying selection, although mild in great extension of the gene [10], [11]. On the other hand, *tra* has been considered to be under strong purifying selection in some species of the *fraterculus* group, although some regions rich in serine-arginine show high levels of polymorphism [79] and the authors failed to perform some specific tests for positive selection, such as the ones performed in PAML. A similar pattern of conserved and variable regions has been reported for another gene from the sex-determining cascade, *fruitless* (*fru*), which has been shown to be conserved at the BTB dimerization and DNA-binding domains, but highly divergent at the N-terminal extension and connecting region [80], where we detected signals of positive selection among species of *Anastrepha* [12].

### Conclusion

Our study shows that although *dsx* is relatively conserved, it has an unusual number of nonsynonymous changes in some domains that were shown to be maintained by positive selection. This might indicate that purifying selection maintains gene function directly related to fitness, but may be not be strong enough to counteract positive selection in other domains of the gene that may be under sexual selection. Therefore, part of the variation found in *dsx* may be explained by adaptive changes promoted by positive selection in regions outside the DNA-binding domain. Furthermore, relaxed selective constraint mediated by pleiotropic effects of this gene across sexes and/or epistatic interaction between *dsx* and the downstream regulated genes could explain the segregation of nonsynonymous substitutions in the rest of the common region of *dsx* and in the male-specific exon.

## Supporting Information

Material S1 **Amino acid alignment of** ***doublesex*** **female isoform used in the divergence-based methods.**
(RTF)

Material S2 **Amino acid alignment of** ***doublesex*** **male isoform used in the divergence-based methods.**
(RTF)

Figure S1 **Plots of** ***pS*** **and** ***pN*** **versus evolutionary distance in nucleotide substitution per nucleotide site.** A) Male isoform. B) Female isoform. *pS* and *pN* stands for proportions of synonymous and nonsynonymous substitutions per synonymous and nonsynonymous sites, respectively.
(TIF)

Table S1 **Sampling locations with geographic, haplotype and species information.** The number in haplotype represents its identification and the letter represent the species at which it was found: O - *A. obliqua*; F - *A. fraterculus*; S - *A. sororcula*; B - *A. bistrigata*; G - *A. grandis*; E - *A. serpentina*; T - *A. striata*. Haplotypes in boldface were obtained from GenBank.
(DOC)

Table S2 **Haplotypes and GenBank accession numbers.**
(XLS)

Table S3 **Amino acid physicochemical properties used in** ***MMO1*** **– TreeSAAP analysis and goodness-of-fit statistic to detect deviation from neutral expectation.** * *p*<0.05, ** *p*<0.01, *** *p*<0.001. NS - Not significant.
(XLS)

Table S4 **Haplotypes, number of haplotypes and polymorphic sites in** ***doublesex*** **.**
(XLS)

Table S5 **Count of haplotypes derived from synonymous and nonsynonymous mutations and ranks for the Mann-Whitney U test.** The counts of derived haplotypes were obtained from the haplotype network estimated for the common region without DM/OD1 domain. Synonymous median = 13; Nonsynonymous median = 22. *Z_c_* corrected for continuity = 1.933; *p* = 0.052.
(XLS)
