# Supplementary material for: Positive and Purifying Selection Influence the Evolution of Doublesex in the Anastrepha fraterculus Species Group
Source: PLoS One. 2012 Mar 13;7(3):e33446. doi: 10.1371/journal.pone.0033446 (PMC3302808; doi:10.1371/journal.pone.0033446)
Supplement: Material S1 — Amino acid alignment of doublesex female isoform used in the divergence-based methods. (RTF) [file pone.0033446.s001.rtf]

Supplementary material S1.
Amino acid alignment of doublesex female isoform used in the divergence-based methods.

                     10        20        30        40        50        60        70        80        90       100                  
            ....|....|....|....|....|....|....|....|....|....|....|....|....|....|....|....|....|....|....|....|
D_ananas_F  MVSEE-NW-NSDTMSDSDMIDSKNDVCGGASSSSGSSISPRTPPNCARCRNHGLKITLKGHKRYCKYRYCTCEKCRLTADRQRVMALQTALRRAQAQDEQ 
D_seche_F   .....-..-.........................................................F................................. 
D_melano_F  .....-..-.........................................................F................................. 
D_erecta_F  .....-..-.........................................................F................................. 
D_virili_F  .....-..-...............................................................D........................... 
D_pseudo_F  .....-..-........................................................................................... 
D_persim_F  .....-..-........................................................................................... 
B_dorsal_F  ....D-..-..........H...A..........................................F.F............................... 
B_oleae_F   ....D-..-..........H...A..........................................F................................. 
B_tryoni_F  ....D-S.-....IA....R...A..........................................F.F............................... 
B_correc_F  ....D-..-..........H...A..........................................F.F....Q.......................... 
C_capita_F  ....D-..-.........IH...A.A........................................F................................. 
A_obliq_F   ....D-..-..........L...A..........................................F................................. 
A_frat1_F   ....D-..-..........L...A..........................................F................................. 
A_frat2_F   ....D-..-..........L...A..........................................F.F............................... 
A_frat3_F   ....D-..-..........L...A..........................................F................................. 
A_frat4_F   ....D-..-..........L...A..........................................F................................. 
A_bistr_F   ....D-..-..........L...A...............................V..........F................................. 
A_grand_F   ....D-..-..........L...A...............................V..........F................................. 
A_serp_F    ....D-..-..........L...A..........................................F................................. 
A_sor_F     ....D-..-..........L...A..........................................F.F............................... 
A_stri_F    ....D-..-..........L...A..........................................F................................. 
M_domest_F  ....DS..HS......T..H.....I...........GT...K......H......K...........F.N........................Q...A 

                    110       120       130       140       150       160       170       180       190       200         
            ....|....|....|....|....|....|....|....|....|....|....|....|....|....|....|....|....|....|....|....|
D_ananas_F  RALHMHEVP---PTTTGTATLLSHH-HHAAAAVAAAPAHVHA-HAHMHGAH---HAAH---G-H-------HSHH-----------GHVLH-HQQ---AA 
D_seche_F   .........PAN.AA--.T-.....-..----...-......H.V.---..---..-.---.G.-------....-----------.....-...---.. 
D_melano_F  .........PAN.AA--.T-.....-..----...-......H.V.---..---..-.---.G.-------....-----------.....-...---.. 
D_erecta_F  .........PAN.AA--.T-.....-..----...-......H.V.---..---..-.---.-.-------....-----------.....-...---.. 
D_virili_F  .S.......P--.AGA-..A.....G..--------------H.V.---..AHS..-.---A-.-----HG.G..-----------A....-...QQA.. 
D_pseudo_F  .........P--.S.S-.TA..G..-..----...-T....TH.V.---P.---.S-.---.-.-----HS....-----------.....Q...---IV 
D_persim_F  .........P--.S.S-.TA..G..-..----...-T....TH.V.---P.---.S-.---.-.-----HS....-----------.....Q...---IV 
B_dorsal_F  .V.QI....P--VVHG-PTA..N..-.L--------------H.-------------.---H-.LNQN-----..----------------------AS. 
B_oleae_F   .V.QI....P--VVHG-PTA..N..-.L--------------H.-------------.---H-.LNQN-----..----------------------AS. 
B_tryoni_F  .V.QI....P--VVHG-PTA..N..-.L--------------H.-------------.---H-.LNQN-----..----------------------AS. 
B_correc_F  .V.QI....P--VVHG-PTA..N..-.L--------------H.-------------.---H-.LNQN-----..----------------------AS. 
C_capita_F  .V.QI....P--GVHA-P.A..N..-.L--------------H.-------------.---H-.LNPN-----..----------------------AT. 
A_obliq_F   .V.Q.....P--VVHA-PTA..N..-.L--------------H.-------------.---H-.LNQN-----..----------------------AT. 
A_frat1_F   .I.Q....SP--VVNA-PTA..N..-.L--------------H.-------------.---H-.LNQN-----..----------------------AT. 
A_frat2_F   .V.Q.....P--VVHA-PTA..N..-.L--------------H.-------------.---H-.LNQN-----..----------------------AT. 
A_frat3_F   .I.Q.....P--VVNA-PTA..N..-.L--------------H.-------------.---H-.LNQN-----..----------------------AT. 
A_frat4_F   .V.Q.....P--VVHA-PTA..N..-.L--------------H.-------------.---H-.LNQN-----..----------------------AT. 
A_bistr_F   .V.Q.....P--VVHA-PTA..N..-.L--------------H.-------------.---H-.LNQN-----..----------------------AT. 
A_grand_F   .V.QT....P--VVHA-PTA..N..-.L--------------H.-------------.---H-.LNQN-----..----------------------AT. 
A_serp_F    .V.Q.....P--VVHA-PTA..N..-.L--------------H.-------------.---H-.LNQN-----..----------------------AT. 
A_sor_F     .V.Q.....P--VVHA-PTA..N..-.L--------------H.-------------.---H-PLNQN-----..----------------------AT. 
A_stri_F    .V.Q.....P--VVHA-PTA..N..-.L--------------H.-------------.---H-.LNQN-----..----------------------AT. 
M_domest_F  .I.Q.....P--VVHP-PTA..NA.-..--------------H.-------------.PLPH-.ITQQLHH.P..PHPHLVDASAV-----------A.. 

                    210       220       230       240       250       260       270       280       290       300         
            ....|....|....|....|....|....|....|....|....|....|....|....|....|....|....|....|....|....|....|....|
D_ananas_F  --------------VAAAAA------APP-SH---SA--------ATAA------SLHGHAHAH----HVHMAAAAAASV--QHQH--H--PHSHH---- 
D_seche_F   A-------------A...-----PS-..A-..LGGPS--------TA.S------.I.......----............A-....QS.--.....---- 
D_melano_F  AA------------A...-----PS-..A-..LGG.S--------TA.S------.I.......----............A-....QS.--.....---- 
D_erecta_F  AA------------A...-----PS-..A-..LGG.S--------TA.T------.........----............A-....QS.PH.....---- 
D_virili_F  TA------------....-----PPPQ..-..LG------------A..HNGSAG.........AHVH.A...SS.V...VQ.Q.Q--.-----..QQQQ 
D_pseudo_F  AS------------G...-----PS-Q..Q..LSGGHNGAGATGP....------.......V.----Q.........T.AH.Q..--.-----QS---- 
D_persim_F  AS------------G...-----PS-Q..Q..LSGGHNGAGATGP....------.......V.----Q.........T.AH.Q..--.-----QS---- 
B_dorsal_F  AAA------------.....AHHHIS-------------------------------------------------------------------------- 
B_oleae_F   AAA------------.....AHHHIS-------------------------------------------------------------------------- 
B_tryoni_F  AAA------------.....AHHHIS-------------------------------------------------------------------------- 
B_correc_F  AAA------------.....AHHHIS-------------------------------------------------------------------------- 
C_capita_F  AAA------------.....AHHHIT-------------------------------------------------------------------------- 
A_obliq_F   AAA------------.....AHHHIS-------------------------------------------------------------------------- 
A_frat1_F   AAA------------.....AHHHMS-------------------------------------------------------------------------- 
A_frat2_F   AAA------------.....AHHHIS-------------------------------------------------------------------------- 
A_frat3_F   AAA------------.....AHHHIS-------------------------------------------------------------------------- 
A_frat4_F   AAA------------.....AHHHIS-------------------------------------------------------------------------- 
A_bistr_F   AAA------------.....AHHHIS-------------------------------------------------------------------------- 
A_grand_F   AAA------------.....AHHHIS-------------------------------------------------------------------------- 
A_serp_F    AAA------------.....AHHHMS-------------------------------------------------------------------------- 
A_sor_F     AAA------------.....AHHHIS-------------------------------------------------------------------------- 
A_stri_F    AAA------------.....AHHHIS-------------------------------------------------------------------------- 
M_domest_F  AAAGVGVGPVPPHHI....-----IP-------------------------------------------------------------------------- 

                    310       320       330       340       350       360       370       380       390       400         
            ....|....|....|....|....|....|....|....|....|....|....|....|....|....|....|....|....|....|....|....|
D_ananas_F  PHHQQPPH---HQHPQQ--Q---QGALRSPPHSDHG----PATSSS-GGAASSSSTTAATSSSGSSS-------NGGAGPGSGAGSAGGGP--------- 
D_seche_F   H...N--.---....H.--.PAT.T...........GSVGA.....G...P...NA..........G-------G..----------...---------- 
D_melano_F  H...N--.---....H.--.PAT.T...........GSVG......G...P...NAA.....N...G-------G..----------...---------- 
D_erecta_F  H...N--.---....H.--.PAT.T...........GSVG..S...G...P...NAV..........-------G..----------...---------- 
D_virili_F  QQ...HH.HSN..Q..PQPH---.AS....S.....GSVSA.....-...-...NVAT.-----...GTAVAG--------------------------- 
D_pseudo_F  HQ..HH..---.....HQP.---HT......PNE.AGSIG......-..G.....GAV.....A...G---AGVG..----------.S.SGSG----SG 
D_persim_F  HQ..HH..---.....HQP.---HT......PNE.AGSIG......-..G.....GAV.....A...G---AGVG.N----------.S.SGSG------ 
B_dorsal_F  ------------------------T.I.....VE.----------------------------------------------------------GGGNVSS 
B_oleae_F   ------------------------T.I.....AE.----------------------------------------------------------GGGNVSS 
B_tryoni_F  ------------------------T.I.....AE.----------------------------------------------------------GGGNVSS 
B_correc_F  ------------------------T.T.....AE.----------------------------------------------------------GGGNVSS 
C_capita_F  ------------------------T.I.....AEL----------------------------------------------------------G----SG 
A_obliq_F   ------------------------T.I....QTE.----------------------------------------------------------G----SG 
A_frat1_F   ------------------------T.I....QTER----------------------------------------------------------G----SG 
A_frat2_F   ------------------------T.V....QTE.----------------------------------------------------------G----SG 
A_frat3_F   ------------------------T.I....QTE.----------------------------------------------------------G----SG 
A_frat4_F   ------------------------T.I....QTE.----------------------------------------------------------G----SG 
A_bistr_F   ------------------------T.I....QTE.----------------------------------------------------------G----SG 
A_grand_F   ------------------------T.I....QTE.----------------------------------------------------------G----SG 
A_serp_F    ------------------------T.I....QTE.----------------------------------------------------------G----SG 
A_sor_F     ------------------------T.I....QTE.----------------------------------------------------------G----SG 
A_stri_F    ------------------------T.I....QTE.----------------------------------------------------------G----SG 
M_domest_F  ------------------------T-I........--------.ANG.------------------------GGG..----------...GGGG----SG 

                    410       420       430       440       450       460       470       480       490       500         
            ....|....|....|....|....|....|....|....|....|....|....|....|....|....|....|....|....|....|....|....|
D_ananas_F  ---RAGS--SG--------------------TSVITSA------------------------------------DHHMT-------------------TV 
D_seche_F   ----G..--..----------GGAGGGRSSG.......------------------------------------.....-------------------.. 
D_melano_F  ----G..--..----------GGAGGGRSSG.......------------------------------------.....-------------------.. 
D_erecta_F  ----G..--..----------GGAAGGRSSG.....T.------------------------------------E....-------------------.. 
D_virili_F  ----...--G.--------------------I......------------------------------------.Q..S-------------------.. 
D_pseudo_F  VGG....--G.--------------------V......------------------------------------.P..S-------------------.. 
D_persim_F  VGG....--G.--------------------V......------------------------------------.P..S-------------------.. 
B_dorsal_F  SGN--.GIAG.IGSAITSVPG---------------.V---------------------------------PPPE....-------------------.. 
B_oleae_F   GGN--.GIAG.IGSGITSVSG---------------.V---------------------------------PPPE....-------------------.. 
B_tryoni_F  SG----GIAG.IGSAITSVPG---------------.V---------------------------------PPPE....-------------------.. 
B_correc_F  TGN--.GIAG.IGSAVTSVPG---------------.V---------------------------------PPPE....-------------------.. 
C_capita_F  GG----GLAG.IGSAITSVPV---------------..---------------------------------PPPE....-------------------.. 
A_obliq_F   GGG--.GMVG.TVPTITSVPV---------------..---------------------------------PPPE....-------------------.. 
A_frat1_F   GGG--.GMVG.TVPTITSVPV---------------..---------------------------------PPPE....-------------------.. 
A_frat2_F   GGG--.GMVG.TVPTITSVPV---------------..---------------------------------PPPE....-------------------.. 
A_frat3_F   GGG--.GMVG.TVPTITSVPV---------------..---------------------------------PPPE....-------------------.. 
A_frat4_F   GGG--.GMVG.TVPTITSVPV---------------..---------------------------------PPPE....-------------------.. 
A_bistr_F   GGG--.GMVG.TVPTITSVPV---------------..---------------------------------PPPE....-------------------.. 
A_grand_F   GGG--.GMVG.TVPTITSVPV---------------..---------------------------------PPPE....-------------------.. 
A_serp_F    GGG--.GMVG.TVPTITSVPV---------------..---------------------------------PPPE....-------------------.. 
A_sor_F     GGG--.GMVG.TVPTITSVPV---------------..---------------------------------PPPE....-------------------.. 
A_stri_F    GGG--.GMVG.TVPTITSVPV---------------..---------------------------------PPPE....-------------------.. 
M_domest_F  SGG--.G--G.-------------------------..GGGSNGGGGGVGPSSSSMNGMASSSSAASSSTAPP--..-.PPDHTHHHHHHHHPHPHLVS. 

                    510       520       530       540       550       560       570       580       590       600         
            ....|....|....|....|....|....|....|....|....|....|....|....|....|....|....|....|....|....|....|....|
D_ananas_F  PTPAQSLEGSCDSSSPSPSSTSGAAI--LPISVSVNRK----------------NGANVPLGQDVFLDYCQKLLEKFRYPWELMPLMYVILKDADANIEE 
D_seche_F   ..........................--..........----------------.............................................. 
D_melano_F  ..........................--..........----------------.............................................. 
D_erecta_F  ..........................--..........----------------.............................................. 
D_virili_F  .......................N.V--......ST..-------------------.....................................G.D.D. 
D_pseudo_F  ..........................--..........----------------.............................................. 
D_persim_F  ..........................--..........----------------.............................................. 
B_dorsal_F  ..........S.T..........---AV.....-.G..P-----------SLHP..V.I..A.....EHR............M...........G.D... 
B_oleae_F   ..........S.T..........---AV.....-.G..P-----------SLHP..V.I..A.....EH.............M...........G.D... 
B_tryoni_F  ..........S.T..........---AV.....-.G..P-----------SLHP..V.I..A.....EH.............M...........G.D... 
B_correc_F  ..........S.T..........---AV.....-.G..P-----------SLHP..V.I..A.....EH.............M...........G.D... 
C_capita_F  ..........S.T..........---AA.....-.G..P-----------SLHP..VHM..A.....EH.............M...........G.D... 
A_obliq_F   ..........S.T..........---AV.....-.G..P-----------PVHP..V.I..A.....EH.............M...........G.D... 
A_frat1_F   ..........S.T..........---AV.....-.G..P-----------PLHP..V.I..A.....EH.............M...........G.D... 
A_frat2_F   ..........S.T..........---AV.....-.G..P-----------PLHP..V.I..A.....EH.............M...........G.D... 
A_frat3_F   .......D..S.T..........---AV.....-.G..P-----------PLHP..V.I..A.....EH.............M...........G.D... 
A_frat4_F   ..........S.T..........---AV.....-.G..P-----------PLHP..V.I..A.....EH.............M...........G.D... 
A_bistr_F   ..........S.T..........---AV.....-.G..P-----------PLHP..V.I..A.....EH.............M.L.........G.D... 
A_grand_F   ..........S.T..........---AV.....-.G..P-----------PLHP..V.I..A.....EH.............M...........G.D... 
A_serp_F    ..........S.T..........---AV.....-.G..P-----------PLHP..V.I..A.....EH.............M...........G.D... 
A_sor_F     ..........S.T..........---AV.....-.G..P-----------PLHP..V.I..A.....EH.............M...........G.D... 
A_stri_F    ..........S.T..........---AV.....-.G..P-----------PLHP..V.I..A.....EH.............M...........G.D... 
M_domest_F  .PT...VDS..............V.VPV.---.-P...PNPEQQQNGADMSI-----------.LI.......I...G....M...........GVD.D. 

                    610       620       630        
            ....|....|....|....|....|....|....|..
D_ananas_F  ASRRIEEGQYVVNEYSRQHNLNIYDGGELRNTTRQCG 
D_seche_F   ..................................... 
D_melano_F  ..................................... 
D_erecta_F  ..................................... 
D_virili_F  ..................N.......----------- 
D_pseudo_F  ..........................----------- 
D_persim_F  ..................................... 
B_dorsal_F  .........H....................S...... 
B_oleae_F   .........H....................S...... 
B_tryoni_F  .........H....................S...... 
B_correc_F  .........H...............R....S...... 
C_capita_F  .........H.............F......S...... 
A_obliq_F   .........H....................S...... 
A_frat1_F   .........H....................S...... 
A_frat2_F   .........H....................S...... 
A_frat3_F   ........PH....................S...... 
A_frat4_F   .........H....................S...... 
A_bistr_F   .........H................S...S...... 
A_grand_F   .........H....................S...... 
A_serp_F    .........H....................S...... 
A_sor_F     .........H....................S...... 
A_stri_F    .........H....................S...... 
M_domest_F  ..K......H................C...CA..... 
