# Supplementary material for: Positive and Purifying Selection Influence the Evolution of Doublesex in the Anastrepha fraterculus Species Group
Source: PLoS One. 2012 Mar 13;7(3):e33446. doi: 10.1371/journal.pone.0033446 (PMC3302808; doi:10.1371/journal.pone.0033446)
Supplement: Material S2 — Amino acid alignment of doublesex male isoform used in the divergence-based methods. (RTF) [file pone.0033446.s002.rtf]

Supplementary material S2.
Amino acid alignment of doublesex male isoform used in the divergence-based methods.

                                         10        20        30        40        50        60        70        80        90       100                  
                                ....|....|....|....|....|....|....|....|....|....|....|....|....|....|....|....|....|....|....|....|
D_ananas_M                      MVSEE-NW-NSDTMSDSDMIDSKNDVCGGASSSSGSSISPRTPPNCARCRNHGLKITLKGHKRYCKYRYCTCEKCRLTADRQRVMALQTALRRAQAQDEQ 
D_seche_M                       .....-..-.........................................................F................................. 
D_melano_M                      .....-..-.........................................................F................................. 
D_erecta_M                      .....-..-.........................................................F................................. 
D_virili_M                      .....-..-...............................................................D........................... 
D_pseudo_M                      .....-..-........................................................................................... 
D_persim_M                      .....-..-........................................................................................... 
B_dorsal_M                      ....D-..-..........H...A..........................................F.F............................... 
B_oleae_M                       ....D-..-..........H...A..........................................F................................. 
B_tryoni_M                      ....D-S.-....IA....R...A..........................................F.F............................... 
B_correc_M                      ....D-..-..........H...A..........................................F.F............................... 
C_capita_M                      ....D-..-.........IH...A.A........................................F................................. 
A_obliq_M                       ....D-..-..........L...A.................................S........F................................. 
A_frat1_M                       ....D-..-..........L...A..........................................F................................. 
A_frat2_M                       ....D-..-..........L...A..........................................F.F............................... 
A_frat3_M                       ....D-..-..........L...A...............................V..........F................................. 
A_frat4_M                       ....D-..-..........L...A..........................................F................................. 
A_bistr_M                       ....D-..-..........L...A..........................................F................................. 
A_grand_M                       ....D-..-..........L...A..........................................F................................. 
A_serp_M                        ....D-..-..........L...A...............................V..........F...A............................. 
A_sor_M                         ....D-..-..........L...A..........................................F.L............................... 
A_stri_M                        ....D-..-D.........L...A..........................................F................................. 
Musca domestica M (AY461854.1)  ....DS..HS......T..H.....I...........GT...K......H......K...........F.N........................Q...A 

                                        110       120       130       140       150       160       170       180       190       200         
                                ....|....|....|....|....|....|....|....|....|....|....|....|....|....|....|....|....|....|....|....|
D_ananas_M                      RALHMHEVP---PTTTGTATLLSHH-HHAAAAVAAAPAHVHA-HAHMHGAH---HAAH---G-H-------HSHH-----------GHVLH-HQQ---AA 
D_seche_M                       .........PAN.AA--.T-.....-..----...-......H.V.---..---..-.---.G.-------....-----------.....-...---.. 
D_melano_M                      .........PAN.AA--.T-.....-..----...-......H.V.---..---..-.---.G.-------....-----------.....-...---.. 
D_erecta_M                      .........PAN.AA--.T-.....-..----...-......H.V.---..---..-.---.-.-------....-----------.....-...---.. 
D_virili_M                      .S.......P--.AGA-..A.....G..--------------H.V.---..AHS..-.---A-.-----HG.G..-----------A....-...QQA.. 
D_pseudo_M                      .........P--.S.S-.TA..G..-..----...-T....TH.V.---P.---.S-.---.-.-----HS....-----------.....Q...---IV 
D_persim_M                      .........P--.S.S-.TA..G..-..----...-T....TH.V.---P.---.S-.---.-.-----HS....-----------.....Q...---IV 
B_dorsal_M                      .V.QI....P--VVHG-PTA..N..-.L--------------H.-------------.---H-.LNQN-----..----------------------AS. 
B_oleae_M                       .V.QI....P--VVHG-PTA..N..-.L--------------H.-------------.---H-.LNQN-----..----------------------AS. 
B_tryoni_M                      .V.QI....P--VVHG-PTA..N..-.L--------------H.-------------.---H-.LNQN-----..----------------------AS. 
B_correc_M                      .VPQI....P--VVHG-PTA..N..-.L--------------H.-------------.---H-.LNQN-----..----------------------AS. 
C_capita_M                      .V.QI....P--GVHA-P.A..N..-.L--------------H.-------------.---H-.LNPN-----..----------------------AT. 
A_obliq_M                       .V.Q.....P--VVHA-PTA..D..-.L--------------R.-------------.---H-PLNQN-----..----------------------AT. 
A_frat1_M                       .I.Q.....P--VVNA-PTA..N..-.L--------------H.-------------.---H-.LNQN-----..----------------------AT. 
A_frat2_M                       .V.Q.....P--VVHA-PTA..N..-.L--------------H.-------------.---H-.LNQN-----..----------------------AT. 
A_frat3_M                       .V.Q.....P--VVHA-PTA..D..-.L--------------H.-------------.---H-.LNQN-----..----------------------AT. 
A_frat4_M                       .V.Q.....P--VVHA-PTA..N..-.L--------------H.-------------.---H-.LNQN-----..----------------------AT. 
A_bistr_M                       .V.Q.....P--VVHA-PTA..N..-.L--------------H.-------------.---H-.LNQN-----..----------------------AT. 
A_grand_M                       .V.Q.....P--VVHA-PTA..N..-.L--------------H.-------------.---H-.LNQN-----..----------------------AT. 
A_serp_M                        .V.Q.....P--VVHA-PTA..N..-.L--------------H.-------------.---H-.LNQN-----..----------------------AT. 
A_sor_M                         .V.Q.....P--VVHA-PTA..N..-.L--------------H.-------------.---H-PLNQN-----..----------------------AT. 
A_stri_M                        .V.Q.....P--VVHA-PTA..N..-.L--------------H.-------------.---H-.LNQN-----..----------------------AT. 
Musca domestica M (AY461854.1)  .I.Q.....P--VVHP-PTA..NA.-..--------------H.-------------.PLPH-.ITQQLHH.P..PHPHLVDVSAV-----------A.. 

                                        210       220       230       240       250       260       270       280       290       300         
                                ....|....|....|....|....|....|....|....|....|....|....|....|....|....|....|....|....|....|....|....|
D_ananas_M                      --------------VAAAAA------APP-SH---SA--------ATAA------SLHGHAHAH----HVHMAAAAAASV--QHQH--H--PHSHH---- 
D_seche_M                       A-------------A...-----PS-..A-..LGGPS--------TA.S------.I.......----............A-....QS.--.....---- 
D_melano_M                      AA------------A...-----PS-..A-..LGG.S--------TA.S------.I.......----............A-....QS.--.....---- 
D_erecta_M                      AA------------A...-----PS-..A-..LGG.S--------TA.T------.........----............A-....QS.PH.....---- 
D_virili_M                      TA------------....-----PPPQ..-..LG------------A..HNGSAG.........AHVH.A...SS.V...VQ.Q.Q--.-----..QQQQ 
D_pseudo_M                      AS------------G...-----PS-Q..Q..LSGGHNGAGATGP....------.......V.----Q.........T.AH.Q..--.-----QS---- 
D_persim_M                      AS------------G...-----PS-Q..Q..LSGGHNGAGATGP....------.......V.----Q.........T.AH.Q..--.-----QS---- 
B_dorsal_M                      AAA------------.....AHHHIS-------------------------------------------------------------------------- 
B_oleae_M                       AAA------------.....AHHHIS-------------------------------------------------------------------------- 
B_tryoni_M                      AAA------------.....AHHHIS-------------------------------------------------------------------------- 
B_correc_M                      AAA------------.....AHHHIS-------------------------------------------------------------------------- 
C_capita_M                      AAA------------.....AHHHIT-------------------------------------------------------------------------- 
A_obliq_M                       AAA------------.....AHHHIS-------------------------------------------------------------------------- 
A_frat1_M                       AAA------------.....AHHHMS-------------------------------------------------------------------------- 
A_frat2_M                       AAA------------.....AHHHIS-------------------------------------------------------------------------- 
A_frat3_M                       AAA------------.....AHHHIS-------------------------------------------------------------------------- 
A_frat4_M                       AAA------------.....AHHHIS-------------------------------------------------------------------------- 
A_bistr_M                       AAA------------.....AHHHIS-------------------------------------------------------------------------- 
A_grand_M                       AAA------------.....AHHHIS-------------------------------------------------------------------------- 
A_serp_M                        AAA------------.....AHHHIS-------------------------------------------------------------------------- 
A_sor_M                         AAA------------.....AHHHIS-------------------------------------------------------------------------- 
A_stri_M                        AAA------------.....AHHHIS-------------------------------------------------------------------------- 
Musca domestica M (AY461854.1)  AAAGVGVGPVPPHHI....-----IP-------------------------------------------------------------------------- 

                                        310       320       330       340       350       360       370       380       390       400         
                                ....|....|....|....|....|....|....|....|....|....|....|....|....|....|....|....|....|....|....|....|
D_ananas_M                      PHHQQPPH---HQHPQQ--Q---QGALRSPPHSDHG----PATSSS-GGAASSSSTTAATSSSGSSS-------NGGAGPGSGAGSAGGGP--------- 
D_seche_M                       H...N--.---....H.--.PAT.T...........GSVGA.....G...P...NA..........G-------G..----------...---------- 
D_melano_M                      H...N--.---....H.--.PAT.T...........GSVG......G...P...NAA.....N...G-------G..----------...---------- 
D_erecta_M                      H...N--.---....H.--.PAT.T...........GSVG..S...G...P...NAV..........-------G..----------...---------- 
D_virili_M                      QQ...HH.HSN..Q..PQPH---.AS....S.....GSVSA.....-...-...NVAT.-----...GTAVAG--------------------------- 
D_pseudo_M                      HQ..HH..---.....HQP.---HT......PNE.AGSIG......-..G.....GAV.....A...G---AGVG..----------.S.SGSG----SG 
D_persim_M                      HQ..HH..---.....HQP.---HT......PNE.AGSIG......-..G.....GAV.....A...G---AGVG.N----------.S.SGSG------ 
B_dorsal_M                      ------------------------T.I.....VE.----------------------------------------------------------GGGNVSS 
B_oleae_M                       ------------------------T.I.....AE.----------------------------------------------------------GGGNVSS 
B_tryoni_M                      ------------------------T.I.....AE.----------------------------------------------------------GGGNVSS 
B_correc_M                      ------------------------T.I.....AE.----------------------------------------------------------GGGNVSS 
C_capita_M                      ------------------------T.I.....AEL----------------------------------------------------------G----SG 
A_obliq_M                       ------------------------T.I....QTE.----------------------------------------------------------G----SG 
A_frat1_M                       ------------------------T.I....QTE.----------------------------------------------------------G----SG 
A_frat2_M                       ------------------------T.V....QTE.----------------------------------------------------------G----SG 
A_frat3_M                       ------------------------T.I....QTE.----------------------------------------------------------G----SG 
A_frat4_M                       ------------------------T.I....QTE.----------------------------------------------------------G----SG 
A_bistr_M                       ------------------------T.I....QTE.----------------------------------------------------------G----SG 
A_grand_M                       ------------------------T.I....QTE.----------------------------------------------------------G----SG 
A_serp_M                        ------------------------T.I....QTE.----------------------------------------------------------G----SG 
A_sor_M                         ------------------------T.I....QTE.----------------------------------------------------------G----SG 
A_stri_M                        ------------------------T.I....QTE.----------------------------------------------------------G----SG 
Musca domestica M (AY461854.1)  ------------------------T-I........--------.ANG.------------------------GGG..----------...GGGG----SG 

                                        410       420       430       440       450       460       470       480       490       500         
                                ....|....|....|....|....|....|....|....|....|....|....|....|....|....|....|....|....|....|....|....|
D_ananas_M                      ---RAGS--SG--------------------TSVITSA------------------------------------DHHMT-------------------TV 
D_seche_M                       ----G..--..----------GGAGGGRSSG.......------------------------------------.....-------------------.. 
D_melano_M                      ----G..--..----------GGAGGGRSSG.......------------------------------------.....-------------------.. 
D_erecta_M                      ----G..--..----------GGAAGGRSSG.....T.------------------------------------E....-------------------.. 
D_virili_M                      ----...--G.--------------------I......------------------------------------.Q..S-------------------.. 
D_pseudo_M                      VGG....--G.--------------------V......------------------------------------.P..S-------------------.. 
D_persim_M                      VGG....--G.--------------------V......------------------------------------.P..S-------------------.. 
B_dorsal_M                      SGN--.GIAG.IGSAITSVPG---------------.V---------------------------------PPPE....-------------------.. 
B_oleae_M                       GGN--.GIAG.IGSGITSVSG---------------.V---------------------------------PPPE....-------------------.. 
B_tryoni_M                      SG----GIAG.IGSAITSVPG---------------.V---------------------------------PPPE....-------------------.. 
B_correc_M                      TGN--.GIAG.IGSAITSVPG---------------.V---------------------------------PPPE....-------------------.. 
C_capita_M                      GG----GLAG.IGSAITSVPV---------------..---------------------------------PPPE....-------------------.. 
A_obliq_M                       GGG--.GMVG.TVPTITSVPV---------------..---------------------------------PPPE....-------------------.. 
A_frat1_M                       GGG--.GMVG.TVPTITSVPV---------------..---------------------------------PPPE....-------------------.. 
A_frat2_M                       GGG--.GMVG.TVPTITSVPV---------------..---------------------------------PPPE....-------------------.. 
A_frat3_M                       GGG--.GMVG.TVPTITSVPV---------------..---------------------------------PPPE....-------------------.. 
A_frat4_M                       GGG--.GLVG.TVPTITSVPV---------------..---------------------------------PPPE....-------------------.. 
A_bistr_M                       GGG--.GMVG.TVPTITSVPF---------------..---------------------------------PPPE....-------------------.. 
A_grand_M                       GGG--.GMVG.TVPTITSVPV---------------..---------------------------------PPPE....-------------------.. 
A_serp_M                        GGG--.GMVG.TVPTITSVPV---------------..---------------------------------PPPE....-------------------.. 
A_sor_M                         GGG--.GMVG.TVPTITSVPV---------------..---------------------------------PPPE....-------------------.. 
A_stri_M                        GGG--.GMVG.TVPTITSVPV---------------..---------------------------------PPPE....-------------------.. 
Musca domestica M (AY461854.1)  SGG--.G--G.-------------------------..GGGSNGGGGGVGPSSSSMNGMASSSSAASSSTAPP--..-.PPDHTHHHHHHHHPHPHLVS. 

                                        510       520       530       540       550       560       570       580       590       600         
                                ....|....|....|....|....|....|....|....|....|....|....|....|....|....|....|....|....|....|....|....|
D_ananas_M                      PTPAQSLEGSCDSSSPSPSSTSGAAI--LPISVSVNRK----------------NGANVPLGQDVFLDYCQKLLEKFRYPWELMPLMYVILKDADANIEE 
D_seche_M                       ..........................--..........----------------.............................................. 
D_melano_M                      ..........................--..........----------------.............................................. 
D_erecta_M                      ..........................--..........----------------.............................................. 
D_virili_M                      .......................N.V--......ST..-------------------.....................................G.D.D. 
D_pseudo_M                      ..........................--..........----------------.............................................. 
D_persim_M                      ..........................--..........----------------.............................................. 
B_dorsal_M                      ..........S.T..........---AV.....-.G..P-----------SLHP..V.I..A.....EH.............M...........G.D... 
B_oleae_M                       ..........S.T..........---AV.....-.G..P-----------SLHP..V.I..A.....EH.............M...........G.D... 
B_tryoni_M                      ..........S.T..........---AV.....-.G..P-----------SLHP..V.I..A.....EH.............M...........G.D... 
B_correc_M                      ..........S.T..........---AV.....-.G..P-----------SLHP..V.I..A.....EH.............M...........G.D... 
C_capita_M                      ..........S.T..........---AA.....-.G..P-----------SLHP..VHM..A.....EH.............M...........G.D... 
A_obliq_M                       ..........S.T..........---AV.....-.G..P-----------PLHP..V.I..A.....EH.............M...........G.D... 
A_frat1_M                       ..........S.T..........---AV.....-.G..P-----------PLHP..V.I..A.....EH.............M...........G.D... 
A_frat2_M                       ..........S.T..........---AV.....-.G..P-----------PLHP..V.I..A.....EH.....G.......M...........G.D... 
A_frat3_M                       ..........S.T..........---AV.....-.G..P-----------PLHP..V.I..A.....EH.............M...........G.D... 
A_frat4_M                       ..........S.T..........---AV.....-.G..P-----------PLHP..V.I..A.....EH.............M...........G.D... 
A_bistr_M                       ..........S.T..........---AV.....-.G..P-----------PLHP..V.I..A.....EH.............M...........G.D... 
A_grand_M                       ..........S.T..........---AV.....-.G..P-----------PLHP..V.I..A.....EH.............M...........G.D... 
A_serp_M                        ..........S.T..........---AV.....-.G..P-----------PLHP..V.I..A.....EH.............M...........G.D... 
A_sor_M                         ..........S.T..........---AV.....-.G..P-----------PLHP..V.I..A.....EH.............M........R..G.D... 
A_stri_M                        ..........S.T..........---AV.....-.G..P-----------PLHP..V.I..A.....EH.............M...........G.D... 
Musca domestica M (AY461854.1)  .PT...VDS..............V.VPV.---.-P...PNPEQQQNGADMSI-----------.LI.......I...G....M...........GVD.D. 

                                        610       620       630       640       650       660       670       680       690       700         
                                ....|....|....|....|....|....|....|....|....|....|....|....|....|....|....|....|....|....|....|....|
D_ananas_M                      ASRRIEEA-------RVEI-------NRIVAQI--------------------------------------------------------YYNYY------ 
D_seche_M                       ........-------....-------..T....--------------------------------------------------------.....------ 
D_melano_M                      ........-------....-------..T....--------------------------------------------------------.....------ 
D_erecta_M                      ........-------....-------..T....--------------------------------------------------------.....------ 
D_virili_M                      ........-------....-------.......--------------------------------------------------------.....------ 
D_pseudo_M                      ........-------....-------.......--------------------------------------------------------.....------ 
D_persim_M                      ........-------....-------.......--------------------------------------------------------.....------ 
B_dorsal_M                      ........KRIVNQT---.SLHWMDR-----.L--------------------------------------------------------.....SSAALV 
B_oleae_M                       ........KRIVNQT---.SLHWMDR-----.L--------------------------------------------------------.....SSAALV 
B_tryoni_M                      ........KRIVNQT---.SLHWMDR-----.L--------------------------------------------------------.....SSAALV 
B_correc_M                      ........KRIVNQT---.SLHWMDR-----.L--------------------------------------------------------.....SSAALV 
C_capita_M                      ........KRIVNQT---.SLHWMDR-----.L--------------------------------------------------------.....SSAALV 
A_obliq_M                       ........KRIVNQT---.SLQLMDR-----.L--------------------------------------------------------.....SSAALV 
A_frat1_M                       ........KRIVNQT---.SLQLMDR-----.L--------------------------------------------------------.....SSAALV 
A_frat2_M                       ........KRIVNQT---.SLQLMDR-----.L--------------------------------------------------------.....SSAALV 
A_frat3_M                       ........KRIVNQT---.SLQLMDR-----.L--------------------------------------------------------.....SSAALV 
A_frat4_M                       ........KRIVNQT---.SLQLMDR-----.L--------------------------------------------------------.....SSAALV 
A_bistr_M                       ........KRIVNQT---.SLQLMDR-----.L--------------------------------------------------------.....SSAALV 
A_grand_M                       ........KRIVNQT---.SLQLMDR-----.L--------------------------------------------------------.....SSAALV 
A_serp_M                        ........KRIVNQT---.SLQLMDR-----.L--------------------------------------------------------.....SSAALV 
A_sor_M                         ........KRIVNQT---.SLQLMDR-----.L--------------------------------------------------------.....SSAALV 
A_stri_M                        ........KRIVNQA---.SLQLMDR-----.L--------------------------------------------------------.....SSAALV 
Musca domestica M (AY461854.1)  ..K.....----------.------------.LFKQYDSLISIYDGHEWRSKASLKRKAESGARNAECDETTKRMRIEATEHLNQLTQT....QRYAAL- 

                                        710       720       730       740       750       760       770       780       790       800         
                                ....|....|....|....|....|....|....|....|....|....|....|....|....|....|....|....|....|....|....|....|
D_ananas_M                      -----------------------------------------------TP-----MAL---VNGAPMYLTYPSIEQSRYGAH---LPFTQIRPPTPEPLAL 
D_seche_M                       -----------------------------------------------..-----...---...............G.....FTH..L...C......... 
D_melano_M                      -----------------------------------------------..-----...---...............G.....FTH..L...C......... 
D_erecta_M                      -----------------------------------------------..-----...---...............G.....FTH..L...C......... 
D_virili_M                      -----------------------------------------------..-----..IGLHTS............--...T.FTH..L............. 
D_pseudo_M                      -----------------------------------------------..-----...---...............G.....FTH..L............. 
D_persim_M                      -----------------------------------------------..-----...---.....................FTH..L............. 
B_dorsal_M                      NTPPTYFPYPIAIGSNGLLTSHF------------------SHLTA-------S.-----------------------------------...S..QPT. 
B_oleae_M                       NTPPTYFPYPIAIGSNGLLTSHF------------------SHLTA-------SI-----------------------------------...S..QPT. 
B_tryoni_M                      NTPPTYFPYPIAIGSNGLLTSHF------------------SHLTA-------S.-----------------------------------...S..QPT. 
B_correc_M                      NTPPTYFPYPIAIGSNGLLTSHF------------------SHLTA-------S.-----------------------------------...S..QPT. 
C_capita_M                      NTVPTYFPYPIAIGSNGLLTSQF------------------SHLTA-------S.-----------------------------------...S..QPT. 
A_obliq_M                       NGPPTYLPYPLAFGTNGLLTSQF------------------SHFTA-------SI-----------------------------------...S..LP.. 
A_frat1_M                       NGPPTYLPYPLAFGTNGLLTSQF------------------SHFTA-------SI-----------------------------------...S..LP.. 
A_frat2_M                       NGPPTYLPYPLAFGTNGLLTSQF------------------SHFTA-------SI-----------------------------------...S..LP.. 
A_frat3_M                       NGPPTYLPYPLAFGTNGLLTSQF------------------SHFTA-------SI-----------------------------------...S..LP.. 
A_frat4_M                       NGPPTYLPYPLAFGTNGLLTSQF------------------SHFTA-------SI-----------------------------------...S..LP.. 
A_bistr_M                       NGPPTYLPYPLAFGTNGLLTSQF------------------SHFTA-------SI-----------------------------------...S..LP.. 
A_grand_M                       NGPPTYLPYPLAFGTNGLLTSQF------------------SHFTA-------SI-----------------------------------...S..LP.. 
A_serp_M                        NGPPTYLPYPLAFGSNGLLTSQF------------------SHFTA-------SI-----------------------------------...S..LP.. 
A_sor_M                         NGPPTYLPYPLAFGTNGLLTSQF------------------SHFTA-------SI-----------------------------------...S..LP.. 
A_stri_M                        NGPPTYLPYPLAFGTNGLLTSQF------------------SHFTA-------SI-----------------------------------...S..LP.. 
Musca domestica M (AY461854.1)  --PPVYWGYP---------SIQFGRAVWTELPNPNFAAIIPPHLAAT..DGPQS---------------------------------------------. 

                                        810       820       830       840       850       860       870       880       890       900         
                                ....|....|....|....|....|....|....|....|....|....|....|....|....|....|....|....|....|....|....|....|
D_ananas_M                      SRSPNSPGGAA------AH-PL---QKHTQSLPQAHSRPGSSNGTVHSAASPTMVTTLATTSSTSTTTLSRRQ---RSRSATPTTPPPPPPAHSNSNGAY 
D_seche_M                       ....S..S.PS------.----AHN..P--------.....................M......P.P......---..................S..... 
D_melano_M                      ....S..S.PS------.----VHN..P--------.....................M......P.--.....---..................S..... 
D_erecta_M                      ....S..S.PS------.----AHN..P--------..........N..........M......P.P......---..................S..... 
D_virili_M                      ..T.--.S-P.AESALGVSN.H---..L--------...A.....A.......L...V..IT-.PG----HH.QQP....G..N...---....S..... 
D_pseudo_M                      ....S..S...------..-.H---...--------....................MMP...-.P.----H..---....G..P..........S..... 
D_persim_M                      ....S..S...------..-.H---...--------....................MMP...-.P.----H..---....G..P..........S..... 
B_dorsal_M                      ..T.--.SPS----------------.P--------.....I---------------------------------------------------------- 
B_oleae_M                       ..T.--.SPS----------------.P--------.....I---------------------------------------------------------- 
B_tryoni_M                      ..T.--.SPS----------------.P--------.....I---------------------------------------------------------- 
B_correc_M                      ..T.--.SPS----------------.P--------.....I---------------------------------------------------------- 
C_capita_M                      ..M.--.SPS----------------.P--------...A.I---------------------------------------------------------- 
A_obliq_M                       ..T.--.SPS----------------.L--------...A.T---------------------------------------------------------- 
A_frat1_M                       ..T.--.SPS----------------.L--------...A.T---------------------------------------------------------- 
A_frat2_M                       ..T.--.SPS----------------.L--------...A.T---------------------------------------------------------- 
A_frat3_M                       ..T.--.SPS----------------.L--------...A.T---------------------------------------------------------- 
A_frat4_M                       ..T.--.SPS----------------.L--------...A.T---------------------------------------------------------- 
A_bistr_M                       ..T.--.SPS----------------.L--------...A.T---------------------------------------------------------- 
A_grand_M                       ..T.--.SPS----------------.L--------...A.T---------------------------------------------------------- 
A_serp_M                        ..T.--.SPS----------------.L--------...A.T---------------------------------------------------------- 
A_sor_M                         ..T.--.SPT----------------.L--------...A.T---------------------------------------------------------- 
A_stri_M                        ..T.--.SPS----------------.L--------...A.T---------------------------------------------------------- 
Musca domestica M (AY461854.1)  ..RS--.SPF----------------.N--------...S..---------------------------------------------------------- 

                                        910       920       930       940         
                                ....|....|....|....|....|....|....|....|....|
D_ananas_M                      ------HHGHHLVSST--------------------------GAT 
D_seche_M                       ------..........--------------------------A.. 
D_melano_M                      ------..........--------------------------A.. 
D_erecta_M                      ------..........--------------------------A.. 
D_virili_M                      HHHHHH...Q......-----------S------AAAVAAAAA.. 
D_pseudo_M                      ------..........--------------------------A.. 
D_persim_M                      ------..........--------------------------A.. 
B_dorsal_M                      -----------.-.E.MSPPAAATNLPS-------SVTAAAA--. 
B_oleae_M                       -----------.-.E.MSPPAAATSLTS-------SATAAAA--. 
B_tryoni_M                      -----------.-.E.MSPPAAATNLPS-------SATAAAA--. 
B_correc_M                      -----------.-.E.MSPPAAAANLPS-------SATATAA--. 
C_capita_M                      -----------.-.D.MSPPATATSLTS-------AATATAA--. 
A_obliq_M                       -----------.-.E.MSPVAATTSLKS-------SATAAAA--. 
A_frat1_M                       -----------.-.E.MSPVAATTSLKS-------SATAAAA--. 
A_frat2_M                       -----------.-.E.MSPVAATTSLKS-------SATAAAA--. 
A_frat3_M                       -----------.-.E.MSPVAATTSLKS-------SATAAAA--. 
A_frat4_M                       -----------.-.E.MSPVAATTSLKS-------SATAAAA--. 
A_bistr_M                       -----------.-.EKMSPAAATTSLKS-------SATAAAA--. 
A_grand_M                       -----------.-.E.MSPVAATTSLKS-------SATAAAA--. 
A_serp_M                        -----------.-.E.MSPVAAATSLKS-------SATAAAA--. 
A_sor_M                         -----------.-.E.MSPVAATTSLKS-------SATAAAA--. 
A_stri_M                        -----------.-.EKMSPAAATTSLKS-------SATAAAA--. 
Musca domestica M (AY461854.1)  -----------.G.ESTT----VTSLPTPGVLAAAAAAAAAAA.. 
