# Supplementary figures and images for: Positive and Purifying Selection Influence the Evolution of Doublesex in the Anastrepha fraterculus Species Group
Source: PLoS One. 2012 Mar 13;7(3):e33446. doi: 10.1371/journal.pone.0033446 (PMC3302808; doi:10.1371/journal.pone.0033446)

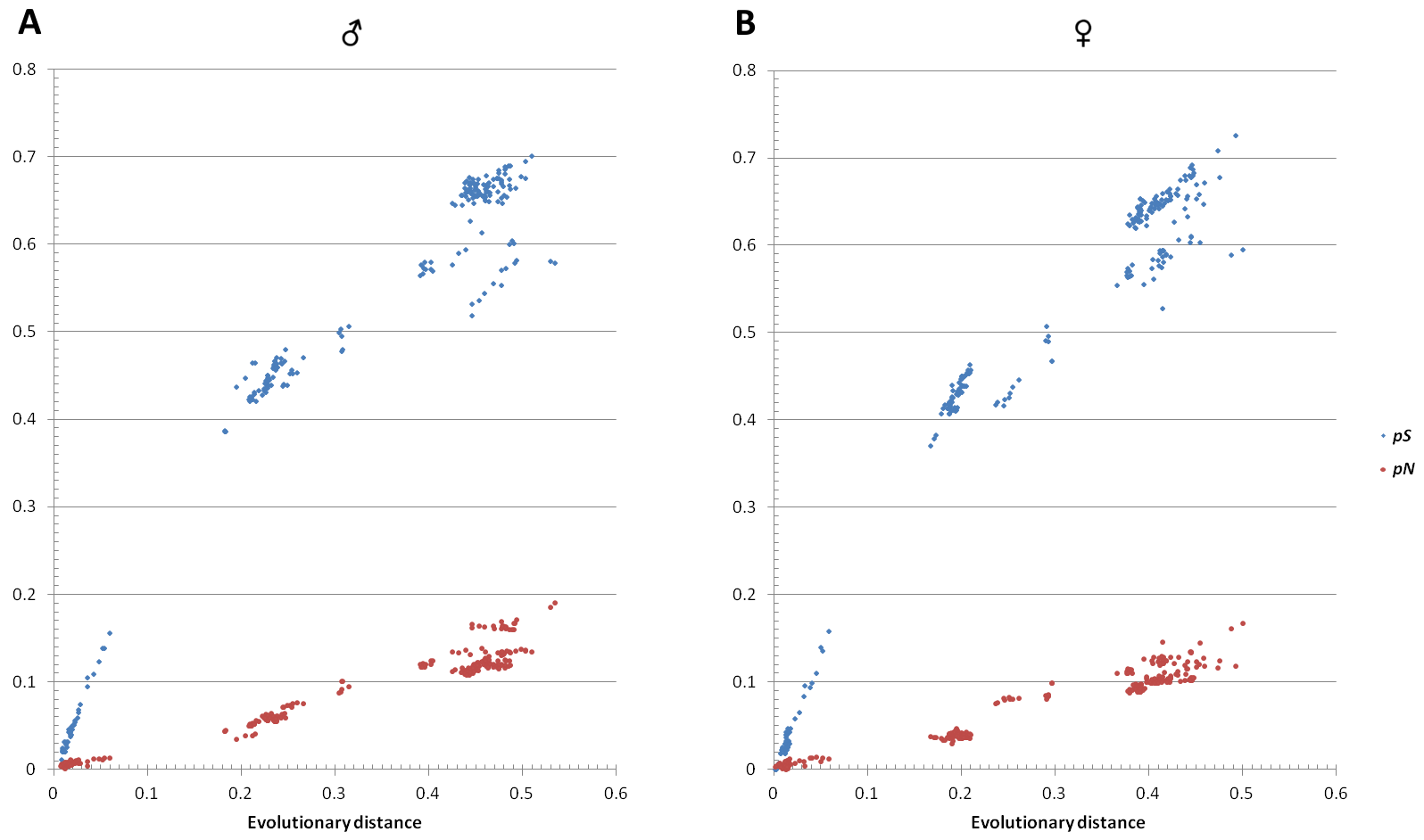

Supplement: Figure S1 — Plots of pS and pN versus evolutionary distance in nucleotide substitution per nucleotide site. A) Male isoform. B) Female isoform. pS and pN stands for proportions of synonymous and nonsynonymous substitutions per synonymous and nonsynonymous sites, respectively. (TIF) [file pone.0033446.s003.tif]
