# Supplementary material for: Positive and Purifying Selection Influence the Evolution of Doublesex in the Anastrepha fraterculus Species Group
Source: PLoS One. 2012 Mar 13;7(3):e33446. doi: 10.1371/journal.pone.0033446 (PMC3302808; doi:10.1371/journal.pone.0033446)
Supplement: Table S1 — Sampling locations with geographic, haplotype and species information. The number in haplotype represents its identification and the letter represent the species at which it was found: O - A. obliqua; F - A. fraterculus; S - A. sororcula; B - A. bistrigata; G - A. grandis; E - A. serpentina; T - A. striata. Haplotypes in boldface were obtained from GenBank. (DOC) [file pone.0033446.s004.doc]

**Table S1. Sampling locations with geographic, haplotype and species information.**

| Code | Collection locality | Latitude  (S) | Longitude  (W) | Haplotype |
| --- | --- | --- | --- | --- |
| 01 | São Carlos - SP | 22° 01' 03'' | 47° 53' 27'' | 9-F |
| 02 | Vitória - ES | 20° 19' 10'' | 40° 20' 16'' | 2-O, 3-O, 25-O, 26-O, 45-O |
| 03 | Feira do Santana -BA | 12° 16' 00'' | 38° 58' 00'' | 4-O, 5-O |
| 04 | Gurupi - TO | 11° 43' 45'' | 49° 04' 07'' | 6-O, 7-O, 41-O, 42-O |
| 05 | São Sebastião – SP (Santiago) | 23° 45' 36'' | 45° 24' 35'' | 8-S, 1-S, 77-F, 78-F, **82-F**,  **92-F, 86-E**, **96-E** |
| 06 | Bertioga - SP | 23° 51' 16'' | 46° 08' 19'' | 10-F, 11-F |
| 07 | Conceição do Almeida - BA | 12° 46' 46'' | 39° 10' 12'' | 12-F, 13-F, 14-F, 15-F |
| 08 | Araguaína – TO | 07° 11' 28'' | 48° 12' 26'' | 16-O, 17-O, 18-O, 19-O,  20-O, 21-O, 22-O |
| 09 | Santo Amaro – BA | 12° 32' 48'' | 38° 42' 43'' | 1-O, 23-O, 24-F |
| 10 | Goiânia - GO | 16° 40' 43'' | 49° 15' 14'' | 27-O, 28-O |
| 11 | Bonito – PE | 08° 28' 13'' | 35° 43' 43'' | 29-F, 1-F, 30-S, 31-S |
| 12 | Redenção – PA | 08° 01' 43'' | 50° 01' 53'' | 32-S, 33-O, 34-O, 35-O |
| 13 | Babaçulândia – TO | 07° 12' 17'' | 47° 45' 25'' | 36-O, 37-O |
| 14 | Pirenópolis – GO | 15° 51' 09'' | 48° 57' 33'' | 38-O, 39-O, 40-F |
| 15 | Três Lagoas – MS | 20° 45' 04'' | 51° 40' 42'' | 43-O, 44-O |
| 16 | Belém -PA | 01° 27' 21'' | 48° 30' 16'' | 46-O, 47-O |
| 17 | Porto Franco - MA | 06° 20' 18'' | 47° 23' 57'' | 48-O, 49-O |
| 18 | Belo Horizonte - MG | 19° 55' 15'' | 43° 56' 16'' | 50-O |
| 19 | Bela Vista de Goiás - GO | 16° 58' 22'' | 48° 57' 12'' | 51-O |
| 20 | Linhares – ES | 19° 23' 28'' | 40° 04' 20'' | 52-O, 53-S, 54-S |
| 21 | Piracicaba – SP | 22° 43' 31'' | 47° 38' 57'' | 55-S, 56-S, 57-S, 58-S |
| 22 | Nova Souré – BA | 11° 14' 00'' | 38° 29' 00'' | 59-S, 60-S |
| 23 | Bauru – SP | 22° 18' 53'' | 49° 03' 38'' | 25-S, 61-S, 62-S, 63-F |
| 24 | Porto Seguro – BA | 16° 26' 59'' | 39° 03' 53'' | 64-S |
| 25 | Boracéia – SP | 22° 11' 35'' | 48° 46' 44'' | 1-F, 65-F |
| 26 | Bariri – SP | 22° 04' 28'' | 48° 44' 25'' | 66-F, 65-F |
| 27 | Itabira – MG | 19° 37' 09'' | 43° 13' 37'' | 67-F, 68-F |
| 28 | Moji das Cruzes – SP | 23° 31' 22'' | 46° 11' 18'' | 69-F |
| 29 | Vacaria – RS | 28° 30' 44'' | 50° 56' 02'' | 70-F, 71-F, 72-F |
| 30 | Vargem Alta – ES | 20° 40' 17'' | 41° 00' 25'' | 73-F, 74-F, 75-F, 76-F |
| 31 | Natal – RN | 05° 47' 42'' | 35° 12' 34'' | 79-F |
| 32 | Caçador - SC | 26° 46' 31'' | 51° 00' 54'' | 80-F |
| 33 | Santa Isabel - SP | 23^o^18' 0" | 46^o^13' 0" | **81-F**, **90-F, 1-F**, **91-F** |
| 34 | Guayaquil - Ecuador | 00^o^02' 0" | 80^o^01' 0" | **83-F**, **93-F** |
| 35 | Parati - RJ | 23^o^10' 0" | 44^o^12' 0" | **87-S**, **97-S** |
| 36 | Indaiatuba - SP | 22^o^42' 0" | 47^o^37' 0" | **19-O**, **89-O** |
| 37 | Laranjal Paulista - SP | 23^o^05´ 0" | 47^o^45' 0" | **85-G**, **95-G** |
| 38 | Ribeirão Preto - SP | 21^o^05' 0" | 47^o^50' 0" | **84-B**, **94-B** |
| 39 | Uberlândia - MG | 18^o^56' 0" | 48^o^13' 0" | **88-T**, **98-T** |

The number in haplotype represents its identification and the letter represent the species at which it was found: O - *A. obliqua*; F - *A. fraterculus*; S - *A. sororcula*; B - *A. bistrigata*; G - *A. grandis*; E - *A. serpentina*; T - *A. striata*. Haplotypes in boldface were obtained from GenBank.
